# Genipap Oil as a Natural Cross-Linker for Biodegradable and Low-Ecotoxicity Porous Absorbents via Reactive Extrusion

**DOI:** 10.1021/acs.biomac.4c00883

**Published:** 2024-10-25

**Authors:** Liliana B. Hurtado, Mercedes Jiménez-Rosado, Maryam Nejati, Faiza Rasheed, Thomas Prade, Amparo Jiménez-Quero, Marcos A. Sabino, Antonio J. Capezza

**Affiliations:** † Department of Chemistry, B5IDA research group, Simon Bolivar University, Caracas 89000, Venezuela; ‡ Fibre and Polymer Technology Department, KTH Royal Institute of Technology, Teknikringen 56, Stockholm SE-10044, Sweden; § Departamento de Química y Física Aplicadas, Universidad de León, Campus de Vegazana, 24007 León, Spain; ∥ Department of Chemistry, KTH Royal Institute of Technology, AlbaNova University Centre, SE-106 91 Stockholm, Sweden; ⊥ Department of Biotechnology, Faculty of Biological Sciences, Quaid-i-Azam University, Islamabad 45320, Pakistan; # Department of Biosystems and Technology, Swedish University of Agricultural Sciences, Box 190, 243 22 Lomma, Sweden; ∇ Division of Industrial Biotechnology, Department of Life Sciences, Chalmers University of Technology, 412 96 Gothenburg, Sweden

## Abstract

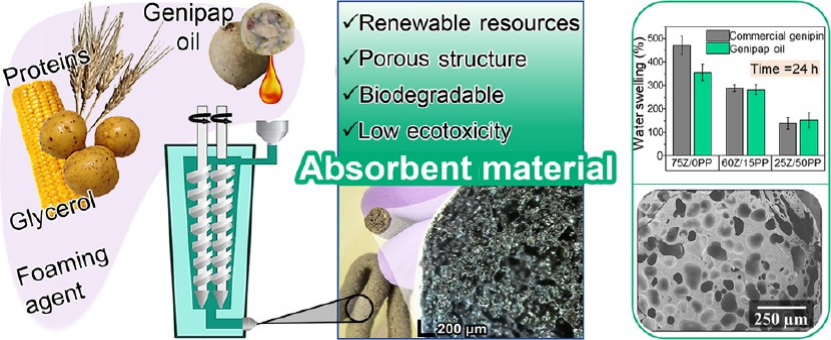

Proteins derived from agroindustrial coproducts and a natural cross-linking agent (genipap oil containing genipin) were used to develop porous materials by reactive extrusion for replacing fossil-based absorbents. Incorporating genipap oil allowed the production of lightweight structures with high saline uptake (above 1000%) and competing retention capacity despite their porous nature. The mechanical response of the genipap-cross-linked materials was superior to that of the noncross-linked ones and comparable to those cross-linked using commercial genipin. The extruded products were hemocompatible and soil-biodegradable in less than 6 weeks. The compounds generated by the degradation process were not found to be toxic to the soil, showing a high bioassimilation capacity by promoting grass growth. The results demonstrate the potential of biopolymers and new green cross-linkers to produce fully renewable-based superabsorbents in hygiene products with low ecotoxicity. The study further promotes the production of these absorbents using low-cost proteins and continuous processing such as reactive extrusion.

## 1 Introduction

Absorbent hygiene products (AHPs), such as baby diapers and sanitary napkins, are widely used because they enhance people’s quality of life.^1−3^ AHPs are single-use, and more than half of their weight is made of synthetic polymers, resulting in poor degradability and even the release of microplastics and toxic molecules such as PFAS.^1−4^ It is estimated that a typical sanitary pad can take up to 800 years to decompose completely, with current methods of disposing of these being incineration and/or landfilling.^1,2^ Quantifying the amount of AHP waste is complex as it depends on the social and economic conditions of the countries.^2,3^ However, estimates in the United Kingdom account that only sanitary napkins generate more than 6,000 tonnes of CO_2_ equivalent GHG annually.^3,4^ For 2019, the world’s average annual production of AHP waste was 45·10^3^ Mt.,^2^ which is almost two times as heavy as The Statue of Liberty.

Using superabsorbent polymers (SAPs) and extruded polyurethane foams (PUR) in AHPs has reduced their weight by 44%, decreasing waste generation.^1,2^ Even so, contamination persists because SAPs have long degradation times or the manufacturing of extruded PUR is highly energy-consuming.^4,5^ Thus, there is growing interest in creating sustainable superabsorbents (SAB) to replace SAPs and PUR in sanitary products. The most acknowledged path to reduce the environmental impact of AHP is to use industrially available raw materials (biopolymers) for their fabrication, minimizing implementation costs while upcycling renewable resources.^4,6−10^ In addition, generating porous structures is the most convenient route to produce absorbing structures as it does not require chemical modifications of the biopolymers, which in turn would elevate their GHG emissions above synthetic counterparts.^11^

Previous studies have shown that using coproducts from the agro-industrial sector, e.g., zein (Z) wheat gluten (WG), as a matrix can potentially reduce the carbon footprint of manufactured bioplastics as porous filaments.^4^ Further, reported thermoplastic materials based on proteins have shown the capacity to absorb and retain large volumes of fluids while exhibiting rapid degradation and high bioassimilation rates.^4−8^ However, a drawback when producing these natural porous structures is poor mechanical properties, requiring chemical cross-linking.^12^ A preferred nontoxic and efficient cross-linking agent of proteins is genipin (GEN),^13^ but its high price would impair any trials of their production on a large scale. Recent studies showed the possibility of using novel genipin-rich extracts known as genipap oil (GO) to cross-link biopolymers at low temperatures.^14^ To our knowledge, no reports of the GO’s potential have been explored using continuous plastic processing techniques such as extrusion. This is unfortunate, as demonstrating the use of GO to cross-link proteins with reactive extrusion while generating a porous absorbent network could represent an economic and feasible route to overcome the limitations of using genipin powder in applications such as AHPs.^14,15^

In this article, we report the first porous cross-linked biopolymer structure produced using plant proteins through reactive extrusion, resulting in relevant functional properties for their use in AHPs. The structures resulted from blending three plant-based proteins, the agroindustry (gluten, zein, and potato) and sodium bicarbonate (SBC) as a foaming agent, glycerol as a plasticizer, and GO as a novel cross-linker. Using denatured industrial potato protein resulted in porous materials with a lower zein content than previously reported, including higher liquid absorption and retention capacity. At the same time, incorporating the inexpensive GO allowed for improved mechanical properties than reference samples and similar properties to samples cross-linked with pure GEN, despite the GO having 10 times less genipin content. Biodegradation and bioassimilation of the samples in soil show that the products are degraded into safe components that can be readsorbed into the next generation of plants. The results revealed that low-temperature extruded porous SABs could be made from industrial biomass and their mechanical properties readily tuned by adding easy-to-handle genipap oil, demonstrating that biobased SABs can be produced more sustainably than current fossil-based SAPs and PUR.

## 2 Experimental Section

### 2.1 Materials

Wheat gluten (WG) was obtained from Lantmännen Reppe AB, Sweden, as a coproduct from industrial wheat starch production. The protein content present in the WG was 86.3 ± 0.3 wt %, determined by the Dumas method, NMLK 6:2003, U.S.A. (N × 6.25). Potato protein concentrate (PP) was supplied by Lyckeby Starch AB, Sweden. In this case, the protein content in PP was 82 ± 2 wt %, which was determined by the Dumas method using a Flash 2000 nitrogen analyzer (N × 6.25). Zein (Z) (C-zein, CAS number: 9010-66-6, product number Z3625) was purchased from Sigma-Aldrich (Sweden). Glycerol (ACS ≥ 99.5% reagent) and sodium bicarbonate (SBC, NaHCO_3_, ACS ≥ 98%) were purchased from Sigma-Aldrich (Sweden). Genipin (GEN) ≥ 98% (HPLC grade) was purchased from Zhixin Biotechnology (China). Genipap oil (GO) was extracted as reported by Hurtado et al., using Genipap (*Genipa americana* L.), a tropical fruit native to Central and South America.^14^ Briefly, maceration of the peeled fruit was followed by a Soxhlet extraction with recirculated chloroform to extract the GO. For the extraction, 1400 g of the peeled fruits were used, resulting in a GO yield of ca. 56 g.

### 2.2 Porous Structures Preparation

**Figure 1:**
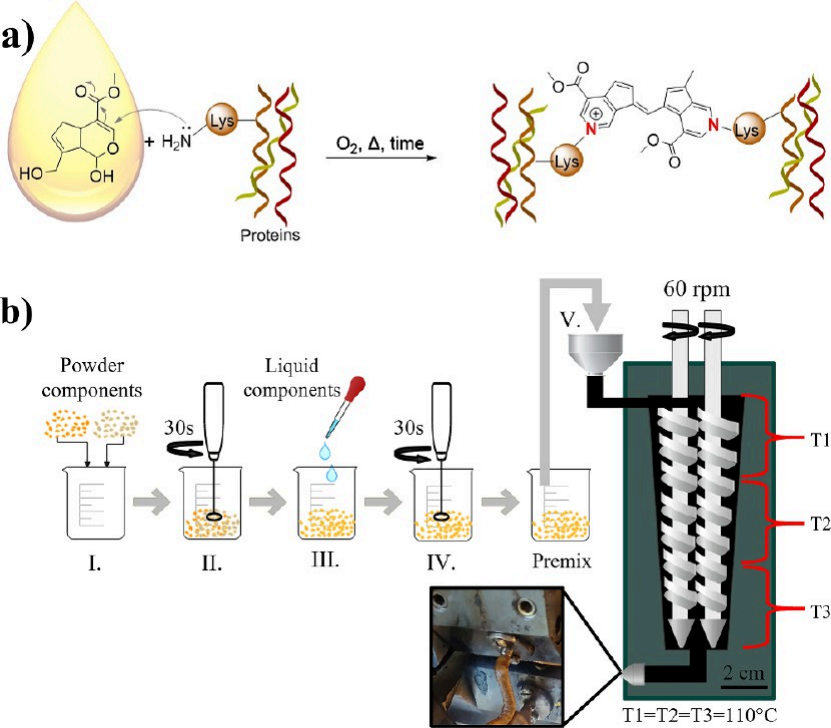
Suggested reaction between protein and genipin (a) scheme of the protocol followed for the preparation of the porous protein structures (b).

Before extruding, the dried Z, WG, and PP powders were mixed at different weight ratios, always having the WG content fixed. The Z:WG:PP mixtures were prepared at the following weight ratios: 75:25:0 (0 wt % PP), 60:25:15 (15 wt % PP), 50:25:25 (25 wt % PP), 40:25:35 (35 wt % PP), 30:25:45 (45 wt % PP) and 25:25:50 (50 wt % PP). SBC was added in the ratio of 5 g/100 g proteins as a foaming agent, glycerol in the ratio of 50 g/100 g proteins as a plasticizer, and GEN and GO in the ratio of 2.5 g/100 g proteins as a cross-linking agent. The selected ratio of SBC and glycerol was selected based on previous studies.^4^ The GEN and GO ratios for the extrusion were selected from a previous study reporting the optimal amount of these, leading to rapid change in the color of biopolymers during thermal treatment in a conventional oven.^14^ The color change is ascribed to cross-linking reactions between GEN and the proteins, and the suggested mechanism is illustrated in Figure 1a. The reaction is based on the nucleophilic attack of the primary amine in proteins (i.e., lysine) to the dihydropyran ring in the genipin, causing its opening and followed by a secondary amine attack on the generated aldehyde group.^16^ The powders were mixed with an electric mixer for 30 s (Figure 1bI.–II.), and glycerol was added and mixed for 30 s to obtain a premix (Figure 1bIII.–IV.). For samples that contained GO, the oil was added together with glycerol (Figure 1bIII.). The premix was immediately transferred to a conical, fully intersecting, and corotating double-screw DSM Xplore 5 cc mini extruder with an L/D ratio of 8 and a compression ratio of 3.3 (The Netherlands). A circular die with a 2.8 mm diameter was used in the extruder. The screw speed was 60 rpm, and all heating zones were set to the same temperature of 110 °C. The extrusion conditions were selected based on previous works that studied protein-based extruded materials at different conditions.^4,14^ The labeling of the extruded samples was assigned based on the Z:PP protein weight ratios and whether they contained a cross-linking agent. For example, the sample prepared with 60 wt % Z, 25 wt % WG, 15 wt % PP, glycerol, SBC, and GEN were named 60Z/15PP^GEN^. The full description of the samples is summarized in Table 1.

**Table 1:** Composition of the Formulations^a^

sample name	Z (wt %)	WG (wt %)	PP (wt %)	GEN (g/100 g protein)	GO (g/100 g protein)
75Z/0PP	75	25	0		
60Z/15PP	60	25	15		
50Z/25PP	50	25	25		
40Z/35PP	40	25	35		
30Z/45PP	30	25	45		
25Z/50PP	25	25	50		
75Z/0PP^GEN^	75	25	0	2.5	
60Z/15PP^GEN^	60	25	15	2.5	
50Z/25PP^GEN^	50	25	25	2.5	
40Z/35PP^GEN^	40	25	35	2.5	
30Z/45PP^GEN^	30	25	45	2.5	
25Z/50PP^GEN^	25	25	50	2.5	
75Z/0PP^GO^	75	25	0		2.5
60Z/15PP^GO^	60	25	15		2.5
50Z/25PP^GO^	50	25	25		2.5
40Z/35PP^GO^	40	25	35		2.5
30Z/45PP^GO^	30	25	45		2.5
25Z/50PP^GO^	25	25	50		2.5

a All formulations contain 50 g glycerol/100 g protein and 5 g SBC/100 g protein.

### 2.3 Thermal Stability

Differential scanning calorimetry (DSC) was used to study the changes in the thermal behavior of the formulations before their extrusion. DSC tests were performed in a Mettler Toledo thermal analyzer (DSC 1) using 100 μL aluminum pan and 10 mg of the formulations. The test was carried out between −10 and 250 °C at a heat rate of 10 °C/min with a nitrogen flow of 50 mL/min. The thermal stability of the porous materials was measured by thermogravimetric analysis (TGA) using a Mettler Toledo thermal analyzer (TGA/DSC 3+) between 30 and 800 °C at a heat rate of 10 °C/min with a nitrogen flow of 50 mL/min.

### 2.4 Density and Apparent Porosity Measurement

The density was calculated by assuming a cylindrical shape for the extruded samples. The apparent density (ρ_a_) was calculated by using the weight of the dry samples (using a Mettler Toledo AL104 analytical balance, USA) divided by its cylindrical volume. Volume dimensions were measured with a digital caliper and 5 specimens per formulation were used. The powder density (ρ_s_) was calculated using eq 1, where *x_i_* is each sample component’s contribution, and ρ_*i*_ is the density of each component. The densities used to estimate the ρ_s_ were as follows: wheat gluten ρ_WG_ = 1300 kg/m^3^,^6^ zein ρ_Z_ = 1220 kg/m^3^,^17^ potato protein ρ_PP_ = 1300 kg/m^3^,^18^ glycerol ρ_G_ = 1260 kg/m^3^,^6^ sodium bicarbonate ρ_SBC_ = 2200 kg/m^3^ (Sigma-Aldrich). GEN or GO were not considered in the calculation due to their low content in the formulations. The extruded material’s apparent porosity was estimated using eq 2, where ρ_a_ is the apparent density of each material, and ρ_s_ is the density of the powders.
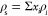
1
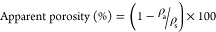
2

### 2.5 Cross-Linking Degree

The degree of cross-linking was determined to evaluate the effect of GEN and GO on the extruded structures. The protocol followed was the one previously reported by Jiménez-Rosado et al.^8^ Briefly, a portion of each sample (15 × 10 × 1 mm^3^) was immersed in a denaturing solution (0.086 mol/L Tris base, 0.045 mmol/L glycine, 2 mmol/L EDTA, 10 g/L sodium dodecyl sulfate (SDS), pH 6 buffer) to extract the noncross-linked protein. The immersion time in the denaturing agent solution was 2 h. Then, the solutions were centrifuged at 10,000*g* for 10 min to separate the denatured protein from the rest of the sample. Finally, the Lowry method was used to estimate the amount of protein in the denatured solution.^19^ The degree of cross-linking was determined using the formulations containing no cross-linker as a reference (0% cross-linking) and a denaturing solution without a sample (100% cross-linking).

### 2.6 Sample Microstructure

The cross-section of the extruded samples was investigated using a Hitachi TM-1000 tabletop SEM (Japan) (10 kV voltage and 6 mm working distance). The extruded samples were immersed in liquid nitrogen for 5 min and cryofractured to evaluate their respective cross-sections. The samples were sputtered with palladium/platinum (Pt/Pd) using an Agar High-Resolution Sputter Coater (model 208RH). The sputtering time for all samples was 30 s × 2 cycles, resulting in an estimated conductive layer of 1–2 nm. The pore size was measured using the image analysis program ImageJ, based on at least 50 measurements (using the 500× magnification images) and reporting the average and standard deviation of the pore sizes. If no pores were possible to measure at 500x, the extruded samples are considered nonporous.

The 3D morphological analysis of the porous materials was assessed via computed X-ray tomography using a YCougar SMT X-ray inspection machine (Yxlon, China). Samples were scanned at an acceleration voltage of 40 kV and a current of 0.1 mA using an open multifocus tube equipped with a tungsten filament. Scans were acquired using a frame rate of 1 s^–1^. Full rotation was used, with projections taken every 0.25°. The 3D analysis was conducted by AVIZO software (ThermoFisher Scientific, USA), binarizing the images by Otsu’s method.^20^

### 2.7 Fourier-Transform Infrared Spectroscopy (FTIR)

The effect of the extrusion processing on the proteins and the addition of the cross-linking agent was studied using FTIR. The equipment used was a PerkinElmer Spectrum 100 with a Graseby Specac Ltd. ATR sensor (England) and a triglycine sulfate (TGS) detector. The spectrum of the samples was obtained with a resolution of 4.0 cm^–1^. Sixteen scans were used in the range of 600–4000 cm^–1^. The samples were kept in a desiccator with silica gel for at least one week before the FTIR measurements.

### 2.8 Liquid Uptake and Retention Capacity

The swelling capacity (SC) in water and limonene of all extruded samples was determined according to a modification of the ASTM D570-98 standard. Cylindrical pieces (1 cm height) were completely immersed in MQw or limonene (used as a hydrophobic liquid to evaluate uptake by capillary effects in porous hydrophilic materials).^6^ Defibrinated sheep blood was also used for the assessment of the absorption simulating a menstrual-like fluid. The immersion time in MQw was 1440 min (24 h), in limonene was 1, 5, 10, 30, and 60 min, and in blood was 1, 5, 10, and 30 min. After the samples were removed from the liquid, excess liquid was removed by placing the samples on tissue paper for 10 s. Next, the sample was weighed and freeze-dried for 24 h at −80 °C to remove all the liquid. SC was calculated using eq 3, and results are reported as the average of triplicates.
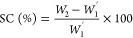
3

*W*_1_′ is the weight of the freeze-dried materials (after swelling) and *W*_2_ is the swollen weight of the extruded sample.

The free liquid swelling (FSC) in a saline solution was determined by a gravimetric method using the Nonwoven Standard Procedure (NWSP) 240.0.R2, also known as the tea bag test. 300 mg of the dry extruded materials, previously ground in liquid nitrogen, was added to a nonwoven fabric bag having a dimension of 40 × 60 mm^2^ (mesh = 400). The bags containing the samples were hooked on a rod and immersed in a beaker containing a 0.9 wt % NaCl solution for simulating body fluids. The immersion times were 1, 5, 10, 30, and 1440 min (24 h), and the working temperature was 25 °C. The bags with the samples were removed from the saline, hung for 10 s out of the solution, and then placed on paper for 10 s to remove unabsorbed saline. Three empty bags were subjected to the same process to obtain an average correction factor (*W*_b_) using eq 4. Finally, the FSC was calculated using eq 5, and the results are reported as the average of triplicates.
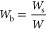
4
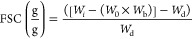
5

*W*_s_ is the weight of the wet blank bag, and *W* is the weight of the dry blank bag. *W*_*i*_ is the weight of the swollen material, *W*_0_ is the dry bag used, and *W*_d_ is the weight of the added dry ground sample. Polyurethane foam (PUR), used as an absorbent core, was manually extracted from a disposable sanitary pad and was used as a reference.

The centrifuge retention capacity (CRC) was assessed to determine the fluid retention of the swollen materials. Approximately 300 mg of the powdered sample was placed in the tea bag and swelled in saline for 30 min, as described in the FSC test. Next, the sample and the tea bag were centrifuged at 1250 rpm for 3 min (according to the NWSP 240.0.R2 standard).^4^ The CRC was obtained using eq 6, where *W*_0_ is the weight of the dry blank bags used, *W*_b_ is the average correction factor, *W*_c_ is the weight of the centrifuged material in the tea bag, and *W*_d_ is the weight of the dry ground sample added. The results were reported from the average of triplicate with their standard deviation.
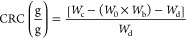
6

### 2.9 Mechanical Properties

Tensile tests were performed by using a universal Instron 5944 testing machine (USA) with a 500 N load cell. The stretch rate was 10 mm/min, and the initial distance between the test machine grips was 10 mm. A series of cylindrical specimens were conditioned at 25 °C and 50 ± 2.5% relative humidity (RH) for 72 h before the analysis. The properties of the wet samples (submerged in MQw at 24 ± 1 °C for 24 h) were also assessed here. Tensile strength, elongation at break, and Young’s modulus were calculated from the stress–strain curve.

A cycle compression test was conducted on random segments of the extruded samples, which were positioned between compression parallel plates (T1223-1021) with a 50 mm diameter and a 500 N load cell in accordance with the ISO 844:2007 standard. The test was performed by using an Instron 5944 universal testing machine (USA). All samples were conditioned at 23 ± 1 °C and 50 ± 2% relative humidity (RH) for at least 120 h prior to the measurement and then cut into 5 mm long cylinders. Samples were compressed in 5 cycles by 30% of their height at 10 mm/min. The samples were allowed to recover for 1 min between the cycles.

Dynamic mechanical analysis (DMA) tests were carried out with a dynamic-mechanical analyzer DMA 850 (Waters, USA) in tensile mode with rectangular geometry. The initial distance between the test machine grips was 10 mm. First, the linear viscoelastic range (interval where the elastic and viscous moduli are independent of the applied strain) was determined by performing strain sweep tests (from 0.002 to 1% strain at 1.0 Hz and room temperature). Second, frequency sweep tests (0.02–20 Hz) were performed within the linear viscoelastic range at room temperature. In these tests, the evolution of the elastic modulus (*E*′), viscous modulus (*E*″), and loss tangent (tan δ) were measured. In addition, elastic modulus and loss tangent at 1.0 Hz (*E*′_1_ and tan δ_1_, respectively) and critical strain (ε_crit_, last strain in the viscoelastic range) were evaluated in order to determine significant differences between the systems. Dry and wet samples were evaluated as in previous tests.

### 2.10 Bioactivity

#### 2.10.1 Hemocompatibility Test

Although this work does not consider the use of the materials in medical devices, where this test is essential for verifying compatibility, it is expected that these materials may come into direct contact with the skin and bodily fluids, such as blood, if they are used in sanitary products. Therefore, it is necessary to perform a simple yet efficient test to assess cytotoxicity. The extruded samples underwent a hemocompatibility test on agar/blood following the protocol established by the ISO 10993-4 (2020) standard. Both sides of the samples were sterilized by ultraviolet (UV) radiation for 60 min before the test. After the samples were sterilized, blood agar cultures using sterile sheep blood were prepared in disposable plates. The samples selected for analysis were 75Z/0PP, 60Z/15PP, and 25Z/50PP from the formulations without a cross-linker, with GEN and GO. The samples were seeded on the plates and placed in an incubator with a flow of 5% CO_2_ at 37 °C (simulating body temperature). Photographic monitoring was done at time zero (moment of implantation of each sample in the blood-agar system) and after 24 h. Acrylamide monomer was used as a hemolytic control, and the blood-agar system alone was used as a nonhemolytic control.

#### 2.10.2 Antimicrobial Activity

In the development of a material with potential applications in hygiene products, SAB must not only have high absorption capacity, water retention, salt resistance, and good mechanical properties but it is also desired to have antimicrobial activity against skin bacteria typical of the mucous membranes of the skin, such as *Escherichia coli* (*E. coli*).^21^ The antimicrobial effect of the extruded samples was measured by the agar diffusion method in triplicates against five bacterial species, namely *Escherichia coli* (CCUG 10979), *Bacillus cereus* (CCUG 7414), *Listeria innocua* (CCUG 15529), *Staphylococcus aureus* and *Pseudomonas aeruginosa*.^22^ Luria agar (LA) plates were used for *E. coli* and *B. cereus*, and TSA (Tryptic Soy Agar) plates were used for *L. innocua*. A solution of OD_600_ = 1 of each bacterial species was prepared and separately spread over the agar plates. The samples were added to the plates after drying, and the plates were investigated for inhibition after 24 h of incubation at 37 °C. Ampicillin (1 μL, 100 mg/ml) and sterile media (1 μL) were used as positive and negative controls, respectively. The antimicrobial activity of the test microorganisms was evaluated by measuring the inhibition zone.

Quantitative antimicrobial activity was determined against gram-positive (*S. aureus*) and gram-negative bacteria (*P. aeruginosa*) and a fungal strain *G. candidum* QAUGC01, using the microdilution technique as described by Seyedain-Ardabili et al.^23^ The bacterial and fungal stains were collected from the Medical Genetics Laboratory, Department of Microbiology, Quaid-i-Azam University, Islamabad. The bacterial and fungal strains were maintained on nutrient agar (NA) and potato dextrose agar (PDA) slants and stored at 4 °C. The nutrient broth (NB) and Potato dextrose broth (PDB) were used for bacterial and fungal growth, respectively. Pure cultures of both bacterial strains were inoculated into the nutrient broth and incubated for 24 h at 37 °C for optimal bacterial growth. An initial population of the bacteria was adjusted according to McFarland to 0.5 units, having a bacterial population of approximately 1.5 × 10^8^ cfu/mL.^24^ The optical density was measured by using a UV/vis spectrophotometer at 600 nm.

The samples were ground, suspended in sterile water, and subjected to serial dilution to determine the minimum inhibitory concentration (MIC). The sample concentration ranged from 500 to 1500 μg (powder material)/mL. 100 μL standard bacterial suspension was added along with 100 μL of different concentrations of the materials in a sterile 96-well microliter plate having nutrient broth. The plate was incubated at 37 °C for 24 h. Negative and positive controls (oxacillin against *S. aureus* and ceftazidime against *S. aeruginosa*) were included. A commercial synthetic pad using PUR foam technology was used as a reference. The MIC for the test organisms was defined as the lowest concentration of the antimicrobial agent that inhibited growth.

#### 2.10.3 Mold Resistance Test

The mold resistance test of the extruded samples is herein performed to evaluate the fungal resistance of the samples at a given temperature and humidity, which simulates extreme storage conditions during shelf life. This test was performed by adding 0.5 g of the extrudates in individual cell culture plates and placing the plates in an airtight container having MQw on the bottom to ensure 100% RH, as seen in Figure S1a. The experiment was carried out at 25 °C and with natural light. Samples are tested without prior sterilization or mold inoculation to simulate real storage conditions. We also assessed the mold resistance of each protein component used in the formulations following the same protocol described above to evaluate correlations between the results and the effect of each additive in the final extrudate. For this, in each well of the culture plates, the proteins were added separately, with each component at a time and including all components, as shown in Figure S1b. The powder and mixing ratios of the additives used are the same as for the extrusion formulations (Table 1). A photographic follow-up was done once a week for 5 weeks.

#### 2.10.4 Biodegradation and Bioassimilation

The material’s soil biodegradability was evaluated by monitoring the decomposition of the sample in contact with the soil over time. The extruded samples were buried in the soil (2:1 farmland:compost) at 20 ± 5 °C temperature and 70–80% RH, according to ISO 20200:2023.^25^ The samples were unearthed on different days for a maximum of 43 days to follow up on their visual appearance and integrity. The test stopped when no pieces larger than 1 mm were found, giving the materials a completely biodegradable classification.

The bioassimilation of the samples was determined by monitoring the germination and growth of grass seeds (*Cynodon dactylon*) in individual parcels. This preliminary test allows us to know whether the samples have a detrimental effect on the soil during its degradation. The soil used was a conventional substrate supplied by COMPO (Spain). The size of the individual parcels was 10 × 10 cm^2^. In each parcel, 0.35 g of sample and 0.5 g of seeds were placed (according to the recommendations from the seed package). The plants were irrigated daily to maintain the soil water saturation limit. A photographic follow-up of the growth of the plants was made at 5, 8, and 12 days after sowing. A control plot without a sample and another with a commercial synthetic pad using PUR foam were used as a reference. After 12 days, the total weight of all the grass (leaf + root) that grew in each parcel was determined, and the results were compared with the controls. Additionally, 20 grass specimens were taken randomly from each parcel, and their leaf and root lengths were measured.

### 2.11 Statistical Analysis

Statistical analysis was performed using variance and Tukey’s HSD posthoc test with a 95% confidence level (*p* < 0.05).^26^

## 3 Results and Discussion

### 3.1 Porous Structures via Reactive Extrusion

**Figure 2:**
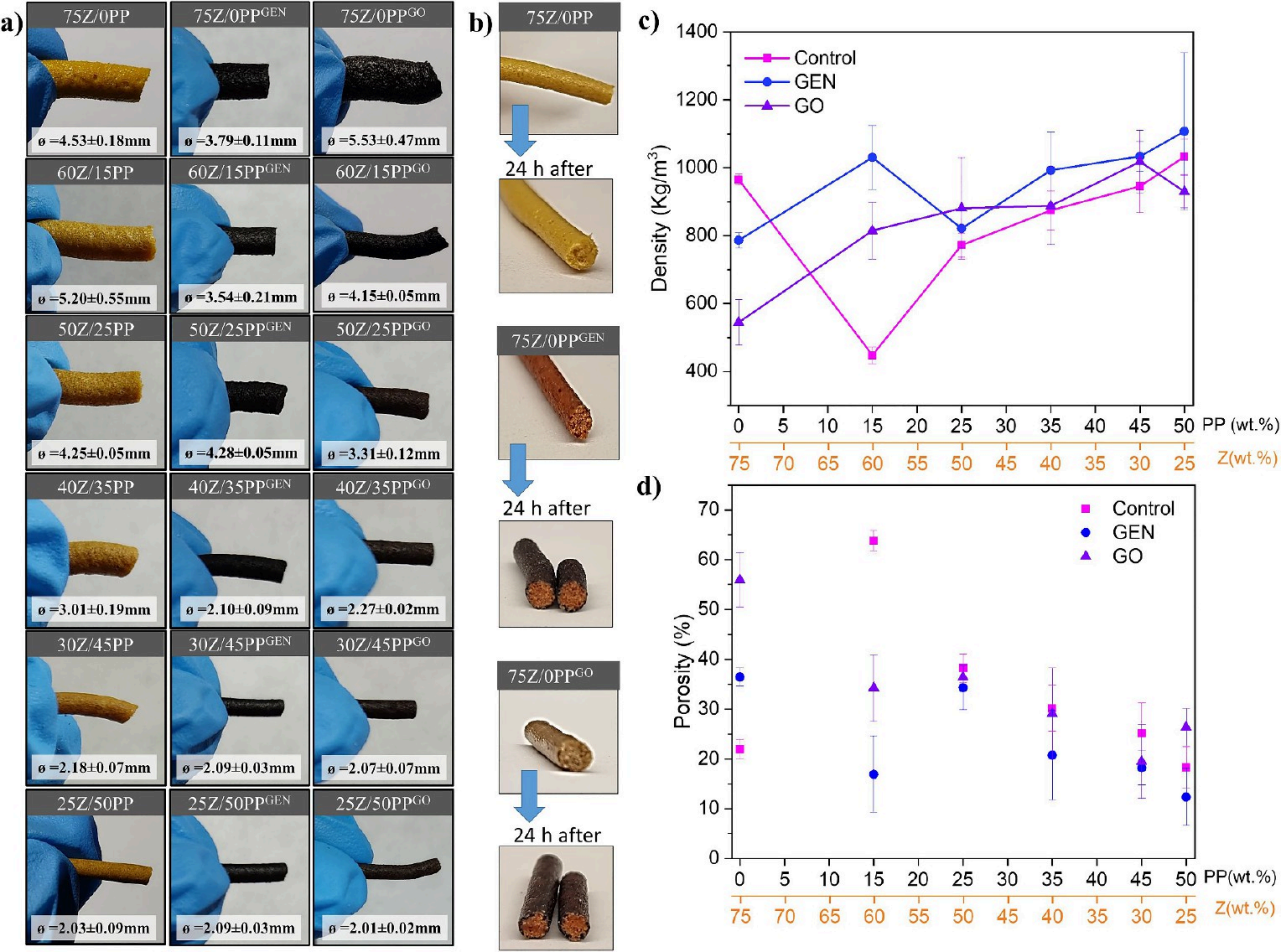
Extruded sample appearance and diameter (⌀) (a), cross-section of the samples immediately after being produced and fractured, and the fracture area after 24 h (b). Apparent density of the extruded samples (c), and calculated porosity using their apparent density (d).

Figure 2 shows that the samples adopt cylindrical shapes with no apparent surface roughness/defects. The main differences observed between the formulations are in the color and expansion ratio. Breaking the extrudates containing GEN or GO after 24 h of extrusion shows a gradient color change from the surface having a darker blue color (Figure 2b). This indicates the absence of oxygen radicals within the sample, as the color change is indicative of a potential reaction mechanism where the radical oxygen from the genipin molecule facilitates cross-linking through the amino group present along the protein chains in each formulation.^22^ The sample with the largest diameter was 75Z/0PP^GO^ with a value of 5.53 mm, where the majority protein is Z, and there is no presence of PP. 25Z/50PP, 25Z/50PP^GEN^, and 25Z/50PP^GO^ had the smallest diameters, about 2.00 mm, with no significant differences among them. The samples with diameters greater than 2.8 mm (the diameter of the circular die used in the extruder) showed an expansion ratio between 1.35 and 2.73, while when the diameter was less than 2.8, the samples showed a compression ratio between 0.26 and 0.79. This phenomenon is known as the die swell or the Barus effect, which is the swelling of a viscoelastic material due to a fast elastic recovery after being subjected to stress. The rapid elastic recovery may be indicative of the fact that the mobility of the polymer chains is not being reduced as a consequence of cross-linking. This effect could generate samples with lower density and high flexibility.^12^

Apparent density and porosity percentage were calculated from the cylindrical shape of the extruded samples (Figure 2c,d). 60Z/15PP had the lowest density of ca. 450 kg/m^3^, while 25Z/50PP^GEN^ had the highest density of ca. 1100 kg/m^3^. Incorporating GEN and GO tended to increase the apparent density, except for the samples without PP. In this case, the apparent density of 75Z/0PP^GO^ and 75Z/0PP^GEN^ was lower than that of 75Z/0PP (Figure 2c). In addition, the trend shows an increase in density with the incorporation of PP, which is ascribed to the samples being less porous with less expansion (see 25Z/50PP, 25Z/50PP^GEN^, and 25Z/50PP^GO^). 60Z/15PP resulted in 64% porosity, and the second highest value was for 75Z/0PP^GO^ with 56% (Figure 2d), these samples being the ones that obtained a higher expansion ratio.

The thermal stability of the blends prior to the extrusion process was measured by DSC. Figure S2 shows a broad endothermic peak at around 100 °C, indicating water evaporation from the protein formulations. Here, the formulations containing 75 wt.% zein (75Z/0PP) shows a *T*_g_ at ca. 155 °C (irrespectively on the presence of GEN or GO), correlating with previous works on zein protein.^27^Figure S2c shows that the formulations start to degrade at around 200 **°**C. Due to the thermal sensibility of protein-based formulations, future studies should focus on more detailed thermal characterization to separate reversible with nonreversible transitions, for example, using DSC in TOPEM mode. During protein extrusion, disulfide bonds are the most common type of covalent cross-linking.^10^ The formation of disulfide bonds (S–S) shows changes in the degree of cross-linking even in the absence of a cross-linker and depends on the type of protein and its composition. The incorporation of PP showed an increase in the cross-linking degree when comparing the samples without a cross-linker (i.e., 60Z/15PP, 50Z/25PP, 40Z/35PP, 30Z/45PP, and 25Z/50PP) with 75Z/0PP, which was used as a reference for 0% cross-linking (Table S1). The overall amino acid profile of PP is low in sulfur-containing amino acids, which are responsible for the formation of disulfide bonds.^7,10,28^ However, PP has a higher lysine content (∼6%) than the average found in most plant-based proteins.^28^ This difference could induce greater branching since the amino group of the side chain has less steric impediment to react.^7^ In fact, lysine is the amino acid most involved in protein cross-linking when a cross-linker such as genipin is used.^7,14,29^

The effect of incorporating GEN and GO in the samples obtained by reactive extrusion was also evaluated by the degree of cross-linking. It is important to mention that the samples of the same protein composition and processed without a cross-linking agent were used as a 0% cross-linking reference. The degree of cross-linking was higher in most samples with GO than with GEN, except for 25Z/50PP^GEN^, which was about 5 times higher than in 25Z/50PP^GO^, as shown in Table S2. The incorporation of GO promotes cross-linking by the presence of genipin (14 wt % genipin) and adds lipids and phenolic compounds that promote covalent and noncovalent bond formation between proteins.^14^ This indicates that the incorporation of the cross-linker in oil form has a beneficial effect on the degree of cross-linking, primarily due to the oil phase’s role during the plasticization stage in the extruder. This facilitates the dispersion between the phases and activates the cross-linking points during melting.^14,30^ However, the decrease in cross-linking degree observed in the samples with GO as PP increased, in contrast to the increase observed in the samples with GEN, suggests that the 14 wt % genipin in GO may be insufficient to cross-link all available amino groups. The cross-linking process requires the formation of genipin dimers in the presence of amino groups to form complete cross-linking.^7,31^ If the ratio of amino groups to genipin is insufficient for genipin dimer formation, complete cross-linking will not occur.^7^

**Figure 3:**
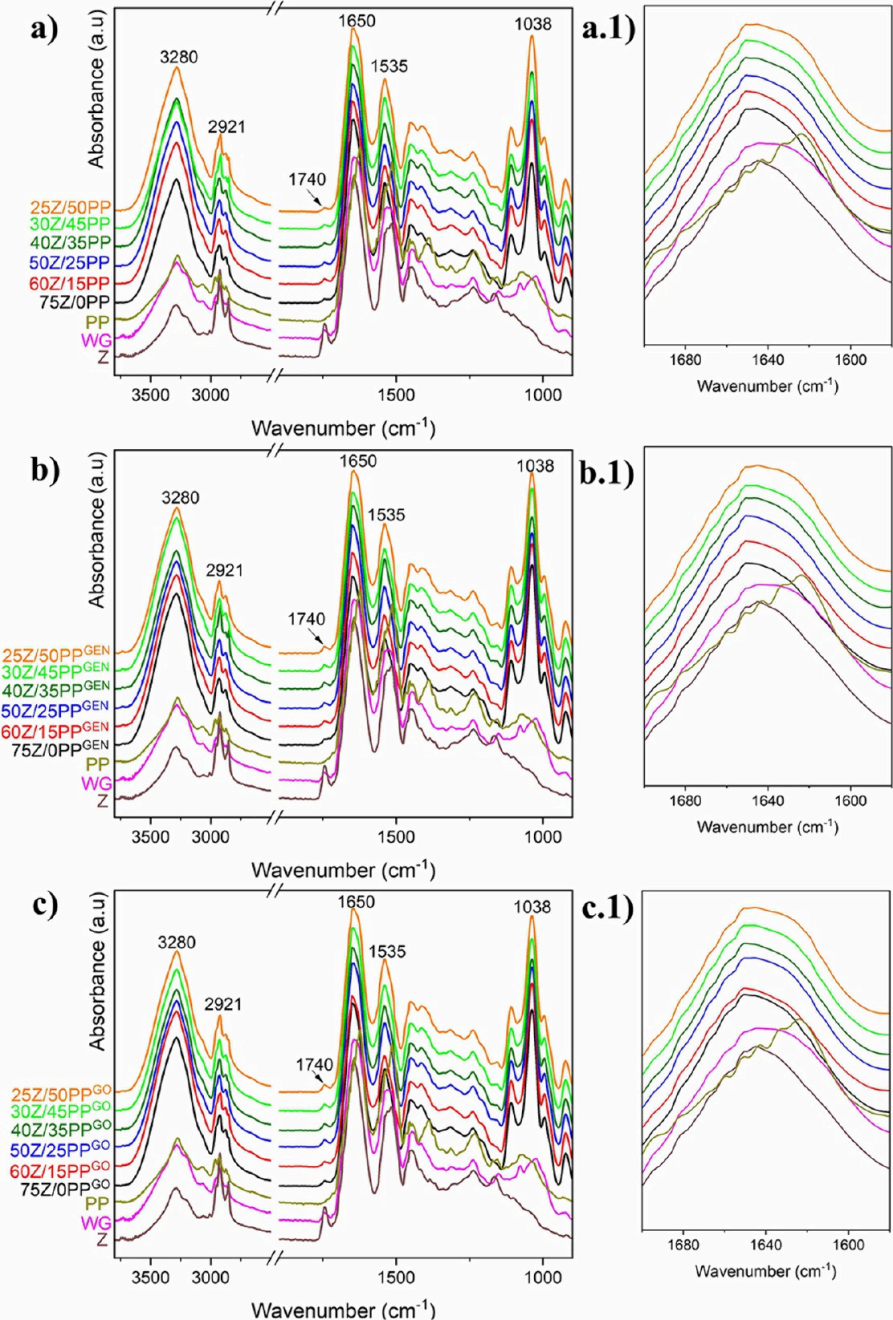
Full FTIR spectra (left) and amide I region (right) of: control formulations (a), formulations with GEN (b), and formulations with GO (c). The spectra of the as-received protein powders are included for better comparison.

The FTIR spectra of the extruded samples and the powder proteins are shown in Figure 3. There was no significant change or shifting in the amine I region (1680–1580 cm^–1^) for the extruded samples as compared to the reference protein powders obtained from the agro-food industry, except for the PP powder. PP shows an amide I profile suggesting a high content of β-sheets, which is the result of the extensive denaturation process performed industrially to precipitate the PP after the potato starch extraction.^32^ It is important to mention that the presence of glycerol in all formulations can prevent changes in the amide I region. Glycerol acts as a plasticizer, increasing the mobility of the chains and reducing their inter/intramolecular interactions.

The absence of evident changes in the amide I region for the cross-linked extrudates is probably due to the small content of GEN or GO. However, the apparent increase in peak intensity in the 1730–1750 cm^–1^ region suggests the presence of carbonyl from the ester group in the genipin molecule (see Figure 3a–c).^7^ When comparing the different samples, the presence of GEN and GO was confirmed in the 1730–1750 cm^–1^ region, being the most intense signal for the samples with commercial GEN (Figure S3).

**Figure 4:**
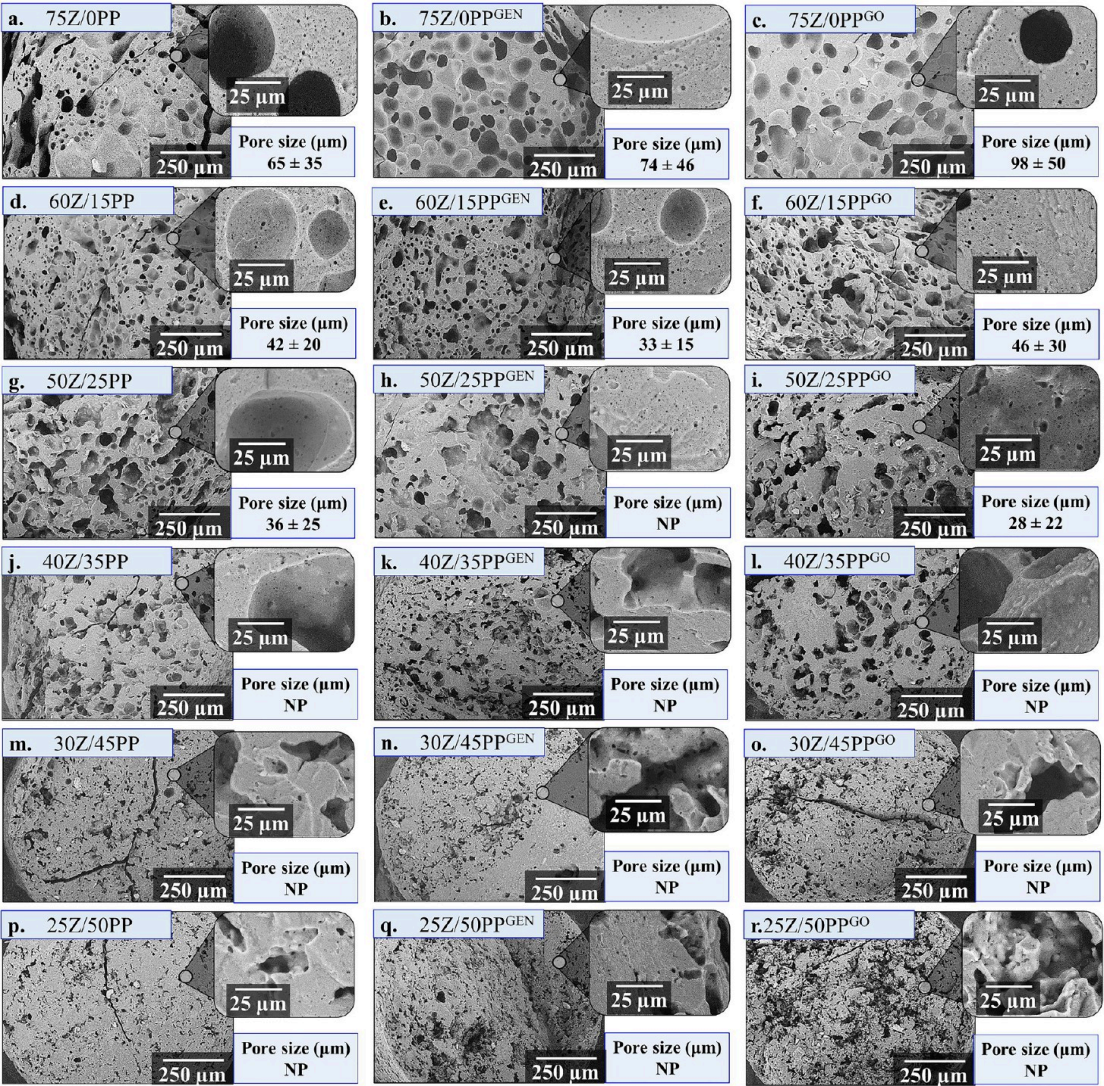
SEM cross-section of the extruded samples processed at 110 °C and 60 rpm. The pore size was measured on 100x SEM images. The sample was considered nonporous (NP) if these were not possible to measure at this magnification.

SEM images of the samples’ cross-section revealed porous structures in the center and edge of the extrudates (Figure 4). The predominance of spherical open pores, especially in 75Z/0PP^GEN^ and 75Z/0PP^GO^, decreased with increasing PP, as shown in Figure 4. Samples with ≥35 wt % PP showed an internal structure with irregularly shaped microcavities, which limited pore size estimation. Overall, the presence of micropores in the cell walls was reduced with increasing PP, independently of the presence of GEN or GO in the composition (Figure 4, high magnification SEM images). The disruption of the pore structure can be correlated to the samples showing a higher cross-linking degree. For instance, the extruded 50Z/25PP^GO^ showed the highest degree of cross-linking (ca. 46%) and was also the sample with the smallest pore size and limited spherical shape (Figure 4i). Previously, it was observed that the degree of cross-linking increases when incorporating PP, even without a cross-linking agent added (Table S1). Furthermore, it is known that protein-based materials with a higher degree of cross-linking show lower porosity,^13^ which can, therefore, collapse the cell structure at the extruder’s die due to elastic effects.

The apparent porosity of 25Z/50PP, 25Z/50PP^GEN^, and 25Z/50PP^GO^ was 18, 12, and 26%, respectively (Figure 2d). This is supported by the dense appearance and lack of pores observed in the SEM images (Figure 4p–r). The generally homogeneous mixtures and extruded structures indicated good mixing between the three proteins and all components in the formulations (Figure 4). However, 40Z/35PP^GO^ (Figure 4l) exhibited large aggregates on the cell walls compared to, for example, 25Z/50PP^GO^ (Figure 4r). The lipids in GO can interact and bind with the hydrophobic sites of proteins during extrusion, which can induce protein aggregation in some preferred proteins. High protein cross-linking through covalent disulfide bonds can also promote protein aggregation.^33^ Here, further fundamental studies are suggested to determine which protein is responsible for the observed effect. The evidence suggests that the effect is caused by the interaction between PP and GO, otherwise not observed in the PP-containing samples having GEN as the cross-linking agent.

Overall, the results reveal that it is possible to produce a porous structure of similar features when using GO instead of GEN. Also, the pores formed when cross-linking the material are relatively more homogeneous and spherical than when the proteins are not cross-linked (compared 75Z/0PP with 75Z/0PP^GEN^ or 75Z/0PP^GO^Figure 4a–c). The use of GO improves the economic feasibility of this type of material vs pure commercial genipin, which is also difficult to disperse in dry extrusion as it has a melting point above 110 °C. This is also the first step toward manufacturing porous materials with reactive extrusion for future green products used in highly consumed applications.

**Figure 5:**
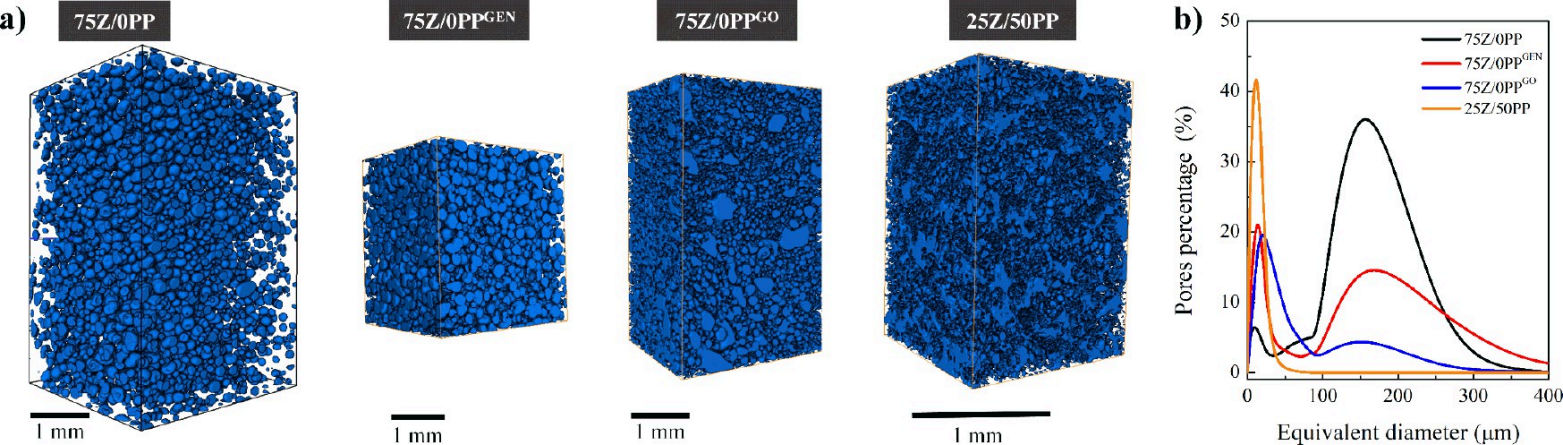
3D rendering of the solid phase for the studied porous protein-based materials (a) and their pore size distribution curves (b).

Figure 5 shows the results of the 3D tomography performed on the extruded samples 75Z/0PP, 75Z/0PP^GEN^, 75Z/0PP^GO^ and 25Z/50PP. The analysis of the extruded samples in Table S3 indicates that 75Z/0PP resulted in the lowest porosity and greatest tendency to form spherical pores, with the pores being concentrated in the central area of the filament and low apparent pore interconnectivity (close cell pores). The addition of the cross-linkers, genipin and genipap oil (75Z/0PP^GEN^ and 75Z/0PP^GO^, respectively), increased the porosity of the systems and allowed for a more open pore structure, although with pore size distributions shifting toward smaller pores than 75Z/0PP (see, Figure 5b). The results could be ascribed to the cross-linker limiting or collapsing the expansion of the pore cell wall surface during extrusion, thus generating smaller pores than the reference system.^4,7,14,15,29^ In addition, the presence of lipids in the GO, which accounts for ca. 70% of its composition, allows greater mobility between chains during the extrusion, resulting in smaller and more interconnected pores.^23^ Moreover, it should be pointed out that the presence of lipids can promote emulsification of the proteins in the formulations. Previous works have already reported that the presence of 1–10 wt % of oil could indeed help to improve the texture and foam stability of porous protein-based materials.^34,35^ Therefore, future work should study the impact of the lipid phase by, e.g., extracting the genipin from the oil and using this oil to evaluate the microstructure. The results show that adding more PP in the formulation (25Z/50PP) impacts the 3D microstructure, with flatter, less spherical, and more interconnected pores compared to 75Z/0PP. The results agree with that observed in the SEM (Figure 4) and the low expansion of 25Z/50PP during processing, with pores having a mean equivalent diameter of 13 μm.

### 3.2 Porous Structures as Liquid Absorbents

**Figure 6:**
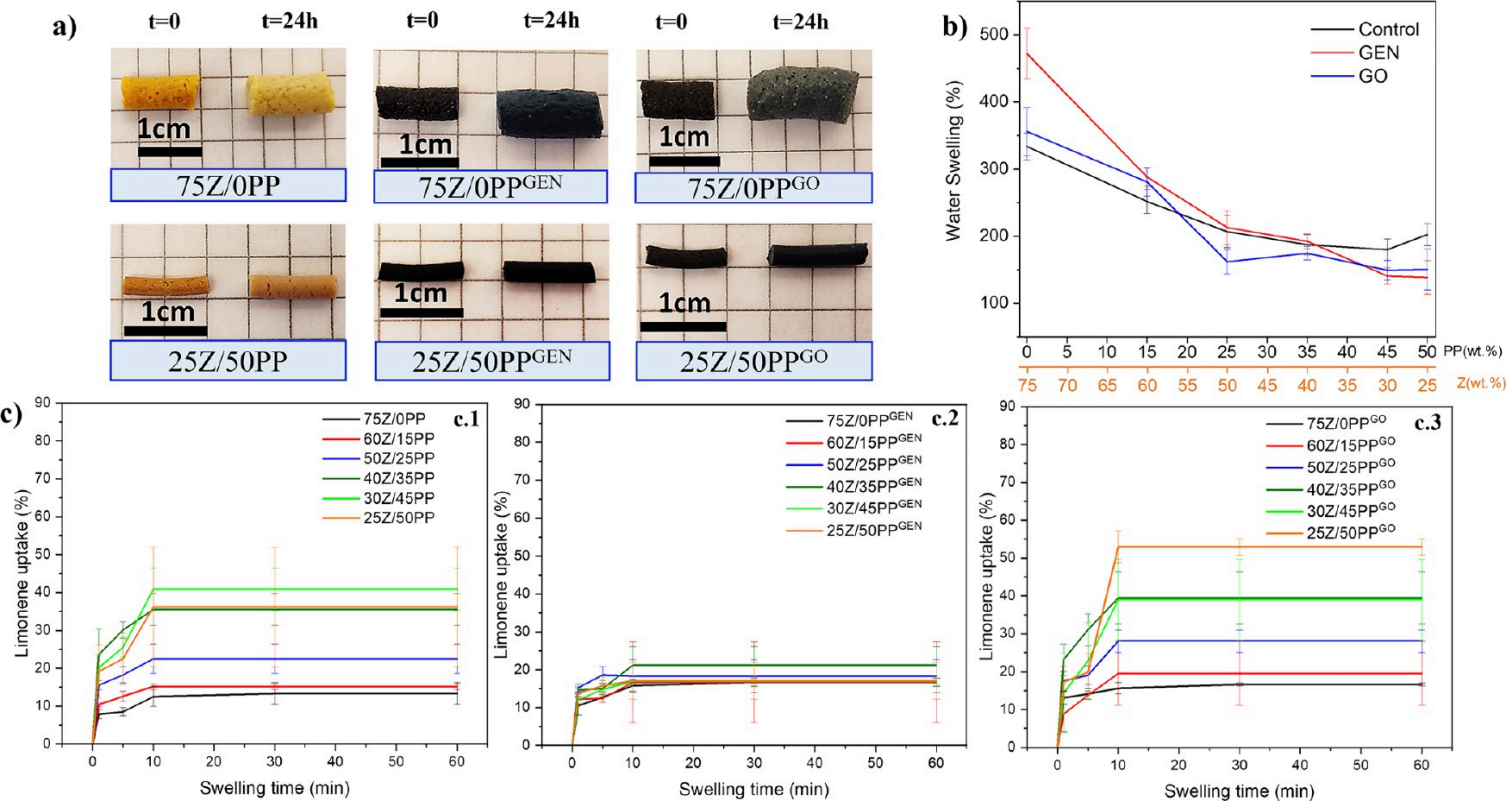
Visual aspect of the dried (left) and 24 h swollen extrudates in MQw (right) (a). Twenty-four hour water swelling capacity of the different extrudates (b). Limonene uptake as a function of time for the extruded controls (c.1), GEN (c.2), and GO samples (c.3).

Figure 6a shows that all samples swelled in MQw after 24 h, keeping high structural stability and retaining their cylindrical shape. This initial observation is of significant interest, as previous studies on protein-based porous structures have demonstrated a loss of structural integrity upon exposure to water.^7,36^ Chen et al. developed foams of WG with glycerol and cross-linked with glutaraldehyde by lyophilization. The study revealed that samples without a cross-linker and those with a low concentration of wheat gluten are unable to maintain their dimensional stability or shape due to insufficient cross-linking.^36^Figure 6b shows the MQw swelling values for all extrudates, with the 75Z/0PP^GEN^ formulation exhibiting the highest swelling at 471%. This agrees with this sample having a higher porosity combined with apparent open and closed pores than the reference (Figure 5a), which improves the diffusion of the liquid and, thereby, its liquid uptake. 75Z/0PP and 75Z/0PP^GO^ both exhibited a MQw swelling of ca. 350%, despite the fact that the porosity of 75Z/0PP^GO^ is higher than that of 75Z/0PP (Figure 5 and Table S3). The swelling capacity of 75Z/0PP^GO^ may have been affected because the samples have flatter pores, which may affect the capillary forces that cause the liquid to be retained in one system more than another or the hydrophobic nature of the sample as a result of the lipid content in the GO.^14^

The extruded samples containing 15 wt % PP (60Z/15PP, 60Z/15PP^GEN^, and 60Z/15PP^GO^) showed a decrease in swelling capacity of about 50% with respect to 75Z/0PP. It was also observed for the other samples that the higher the PP content, the lower the swelling capacity. The swelling values obtained for 60Z/15PP, 60Z/15PP^GEN^, and 60Z/15PP^GO^ were similar at 250, 288 and 280% respectively. In this case, the presence of GEN or GO increased the swelling capacity by 30%, with no significant difference in swelling capacity among them.

The lowest swelling was for 25Z/50PP^GEN^, with a value of 139% compared to 151 and 202% for 25Z/50PP^GO^ and 25Z/50PP, respectively. Although the differences were not significant, the greater cross-linking observed for 25Z/50PP^GEN^ (Table S2) could have caused a decrease in swelling. A higher degree of cross-linking forms a denser protein network, as seen by SEM in Figure 4q and 3D tomography in Figure 5a, making it more difficult for the liquid to diffuse and swell inside the protein cell wall. Thus, according to the maximum swelling obtained, the most promising formulations for the extrusion of cross-linked porous superabsorbent materials are 75Z/0PP^GEN^, 75Z/0PP^GO^, 75Z/0PP, 60Z/15PP^GEN^, 60Z/15PP^GO^ and 60Z/15PP (ordered from greater to lesser capacity to retain water).

The highest swelling sample reported here (471% for 75Z/0PP^GEN^) equals the swelling capacity of similar materials where glutaraldehyde was used as a cross-linker and obtained by lyophilization.^6^ The use of genipin and GO is herein a promising alternative for the development of environmentally friendly superabsorbent materials, as previous works have shown that these alternative cross-linkers are not toxic.^14,37^ Furthermore, for large-scale applications, reactive extrusion is a more continuous and faster process than lyophilization, making it more easily scalable.

The uptake capacity of the extrudates in a nonpolar liquid (limonene) was also evaluated, as limonene does not interact with the proteins and provides information about the capillarity uptake, which is of relevance in foam-based absorbents.^38^Figure 6 shows the extrudates have a rapid uptake at short immersion times (1 and 5 s), reaching a plateau after 10 min of immersion. The highest limonene uptake was achieved in the 25Z/50PP^GO^ sample with a value of 53%, while the lowest was 75Z/0PP^GEN^, 75Z/0PP^GO^, and 75Z/0PP with values of 14, 17, and 19%, respectively. The reason for the low uptake of limonene in the later samples is probably due to their large pores/channels (Figure 4a–c), where high capillary action is obtained in materials with smaller pores.

The effect of the inclusion of GEN or GO in the formulations with protein ratios 75Z/0PP, 60Z/15PP, and 50Z/25PP did not show a significant difference. However, for the 40Z/35PP, 30Z/45PP and 25Z/50PP ratios, there was a decrease (∼19%) in limonene uptake with the use of GEN (Figure 6c.2) and an increase (∼10%) in limonene uptake with the use of GO (Figure 6c.3), both with respect to the control samples (Figure 6c.1). This suggests that lipids in GO have a positive effect on nonpolar fluid uptake.

For all samples, the maximum limonene uptake was 3–6 times lower than that of water (Figure 6b,c), similar to that reported for wheat gluten biofoams.^38^ Although water and limonene have different viscosities (i.e., 0.891 and 0.923 mPa·s,^39^ respectively) and polarities that can affect the diffusion process within the porous structure, the limonene uptake in these biobased materials is related to sole capillary actions. The capillary action is demonstrated by most samples reaching 40–60% of their maximum limonene uptake within 1 min, in contrast to what is observed for water uptake.

**Figure 7:**
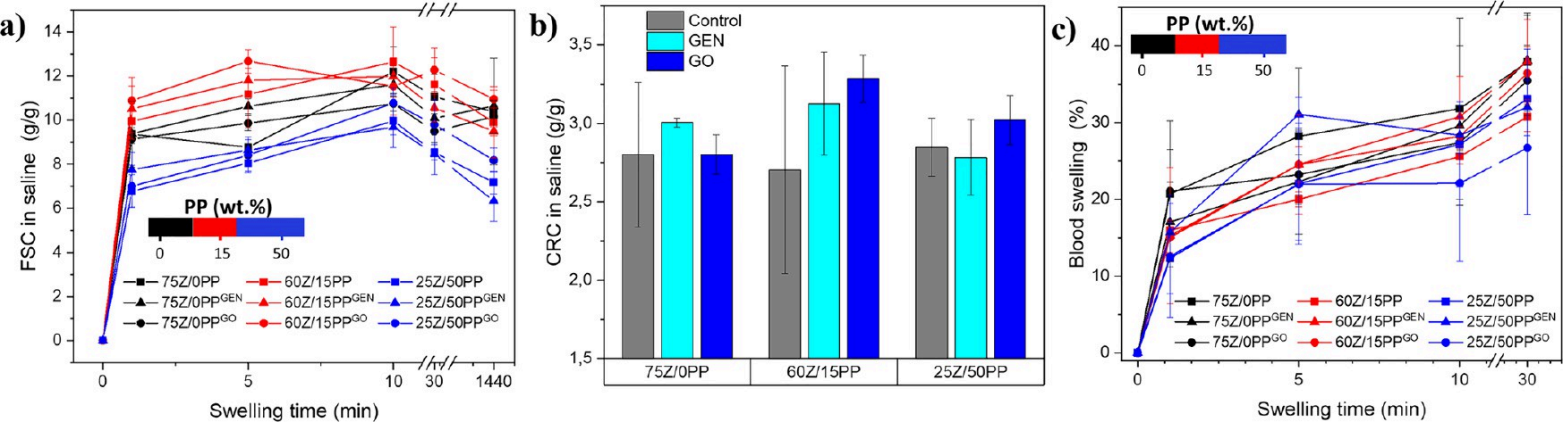
Saline solution (0.9 wt % NaCl) free swelling capacity (FSC) of the ground extrudates (a) and their centrifuge retention capacity (CRC) after 30 min FSC in saline and spun at 1230 rpm for 3 min (b). Swelling capacity of the extrudates in defibrinated sheep blood, used as reference fluid for menstruation sanitary pads (c).

Free swelling capacity (FSC) in saline solution was carried out as a relevant functional property for materials to be used as absorbents in sanitary products. The FSC on ground extrudates was measured only on those samples that resulted in the highest swelling/uptake of MQw or limonene, as shown in Figure 7a. Here, all samples exhibited rapid saline absorption in the first 60 s, the highest being for 60Z/15PP^GO^, 60Z/15PP^GEN^, and 60Z/15PP with a value of ∼10 g of saline per gram of powdered sample (g/g), and the lowest being for 25Z/50PP^GO^, 25Z/50PP^GEN^ and 25Z/50PP with ∼7 g/g (Figure 7a). After 24 h, 60Z/15PP^GO^, 60Z/15PP and 60Z/15PP^GEN^ reached FSC values of 11, 10 and 9 g/g, respectively. Similarly, 75Z/0PP, 75Z/0PP^GEN^, and 75Z/0PP^GO^, which had larger pore sizes when extruded (Figure 4a–c) and MQw swelling >300% (Figure 6b), reached an FSC in saline of ca. 10 g/g. The incorporation of 15% PP increased the FSC with respect to the samples without PP. However, increasing the PP content further results in a decrease in FSC at 5 min, regardless of the presence of a cross-linker (Figure S4). 25Z/50PP^GO^ was found to increase FSC by 1 and 2 g/g over 25Z/50PP and 25Z/50PP^GEN^, respectively. This suggests that GO can be used as a cost-effective alternative to GEN without compromising FSC and the mechanical integrity of the samples. Here, it is worth mentioning that protein-based materials immersed in aqueous liquid have a complete loss of glycerol after 24 h.^4,15^ Thus, the sample that absorbed the most saline here, i.e., 60Z/15PP^GO^ (Figure 7a), would have a theoretical uptake of ca. 16 g/g (instead of 11 g/g), which is, to the best of our knowledge, the highest value reported for porous protein-based materials using reactive extrusion.

Poly(acrylic acid) SAPs are commonly used in commercial diapers due to their high saline solution absorbance (ca. 50 g/g).^40^ Here, the PUR foam pads used as a reference had a saline solution capacity of about 20 g/g after 30 min of swelling. As shown, 60Z/15PP^GO^ achieved a theoretical FSC of 16 g/g in saline, which is nearly 80% of the capacity of the pad reference. The lower absorption values could be attributed to a limited interaction of the liquid with the hydrophobic groups present in the proteins, the osmotic pressure generated, and the charge repulsion between the liquid ions and the protein groups.^28^

Centrifuge retention capacity (CRC) is also reported here as a critical property for assessing materials to be used in sanitary applications (Figure 7b). The 60Z/15PP^GO^ sample exhibited the highest CRC value of 3.3 g/g, indicating that ca. 33% of the saline solution was strongly retained within the network. The water retained in the network is commonly referred to as bound water or half-bound water. This is because free water in a hydrogel has high mobility and can be easily lost during centrifugation.^41^ However, 25Z/50PP^GEN^ retained ca. 44% of saline despite having the lowest absorption capacity among all samples studied at 6 g/g. All in all, the CRC results are comparable with those reported for synthetic SAPs, which achieved a retention of ∼60% in saline.

Figure 7c shows the swelling capacity of the extruded samples in defibrinated sheep blood, indicating the potential of these materials for their use as absorbents in disposable menstruation pads. The highest blood absorption was for the 75Z/0PP, with 21% at 60 s, and the lowest was 12% for 25Z/50PP. After 30 min, the 75Z/0PP, 75Z/0PP^GEN^, and 60Z/15PP^GEN^ samples reached ∼38% blood absorption. The low blood uptake capacity of the samples compared to that of water can be related to the limited diffusion capacity of the blood, reporting a viscosity between 3.5 and 5.5 cP at a temperature of 37 °C,^42^ while the viscosity of water at the same temperature is 0.692 cP. Future studies should focus on obtaining materials with larger pore sizes that are tailored for more viscous liquids and/or ground the materials to favor more permeable surfaces for blood diffusion. However, the rapid saline and high FSC and CRC suggest the material can be used in disposable diapers.

### 3.3 Mechanical Properties

**Figure 8:**
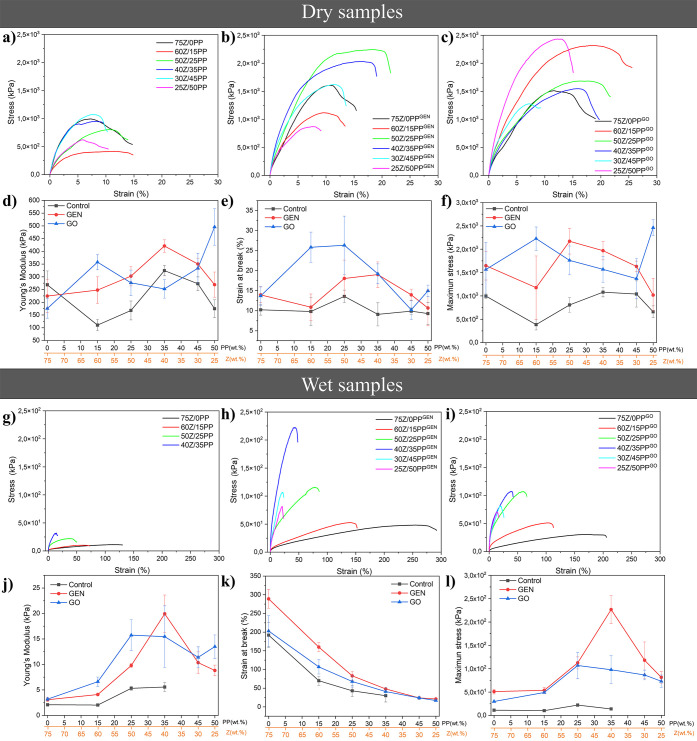
Representative tensile stress–strain curves of the extruded samples: control (a), GEN (b), and GO (c). Summary of their average Young’s modulus (d), strain at break (e), and maximum stress (f) of the different dry extruded samples. Representstive tensile stress–strain curves for the wet extruded samples soaked in MQw for 24 h at 25 °C: control (g), GEN (h), and GO (i). Summary of the average Young’s modulus (j), strain at break (k), and maximum stress (l) of the different wet samples.

The results of the tensile evaluation of the dry and wet extruded samples are listed in Figure 8. The addition of PP did not have a direct correlation with the tensile strength in the samples that did not contain a cross-linking agent (Figure 8a). Further, Young’s modulus does not show a consistent trend with the increasing amount of PP wt % in any of the systems studied (Figure 8d). The samples containing GEN and GO exhibited a tensile strength three times higher than that of similar formulations without a cross-linker (Figure 8a–c). The incorporation of GEN and GO as cross-linking agents also increased the strain and stress at break compared to the reference samples, thus improving the mechanical properties of these cross-linked porous materials (Figure 8d–f). However, the samples with low PP content (e.g., 60Z/15PP^GO/GEN^ and 50Z/25PP^GO/GEN^) showed a 10–15% increase in strain compared to the control, while the strain at break was higher for the GO than for the GEN systems. The results support that GO could cross-link the protein-based material and partially plasticize the proteins. The Young’s moduli of cross-linked materials by GO were mostly lower than those of genipin, although the actual amount of genipin contained in the GO is lower, as shown in Figure S5. The result can be attributed to an increase in the degree of cross-linking, as demonstrated in Tables S1 and S2. Despite the difference in the actual genipin concentration between the GEN and GO cross-linked samples, with the GO used having only 14% of genipin,^14^ the mechanical properties still showed the effectiveness of GO as a cross-linking alternative to commercial GEN for reactive extrusion of agro-industrial proteins.

Figure 8g–i shows that stretching the wet extruded samples reduced the stiffness and maximum stress by 2–3 orders of magnitude compared to the dry samples (Figure 8a–c), indicating the plasticizing effect of water.^4^ All samples maintained their shape in water, as shown in Figure 6a. However, the 30Z/45PP and 25Z/50PP samples fragmented when manipulated and were not tested here. The wet instability of these wet extrudates may be due to the cross-linking of the polymer chains resulting from the presence of 45–50 wt % PP. These samples reached a 56.8 and 54.8% cross-linking degree, respectively, compared to the reference 75Z/0PP (Table S1). It is important to note that the results are a function of the swelling capacity of each sample rather than a fixed water content, which complicates the interpretations. Nonetheless, the results show that increasing the PP content increases the elastic modulus and decreases the stress and maximum elongation, irrespective of the use of GEN or GO (Figure 8g–l).

Regarding the cyclic compression tests, they were performed for the systems 75Z/0PP, 75Z/0PP^GEN^, 75Z/0PP^GO^, and 25Z/50PP, which were the best-performing materials for uptake capacity. Figure S6 shows that all of the protein-based extrudates do not have a complete recovery like the synthetic commercial PUR pad, which had a small hysteresis loop size. The result is ascribed to the synthetic materials having a more elastic network than the herein-produced materials. However, it is worth noticing that the hysteresis loop size for the protein-based materials that included GEN and GO is smaller than the control sample, which is a further elucidation of the cross-linking reactions taking place in the protein.

**Figure 9:**
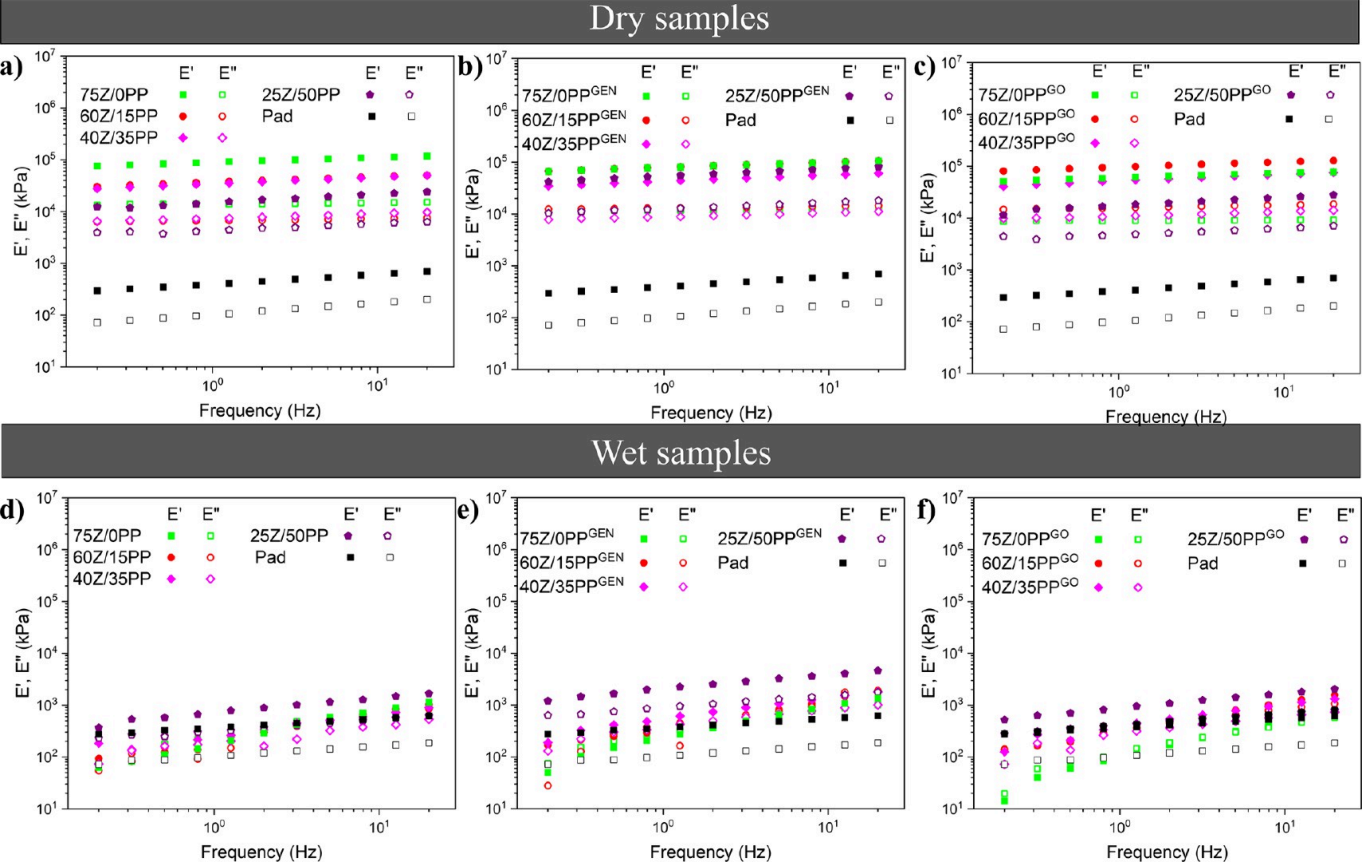
Elastic (*E*′) and viscous (*E*″) moduli vs frequency in the dynamic tensile tests for the dry extruded samples: (a) control, (b) with GEN and (c) with GO; and the soaked samples in saline solution (0.9 wt % NaCl) for 24 h: (d) control, (e) with GEN and (f) with GO.

Figure 9 shows the behavior of dry and wet samples in the tensile dynamic analysis. Generally, all dry samples (Figure 9a–c) had low variations of the elastic (*E*′) and viscous (*E*″) moduli with frequency changes. This behavior correlates with the high structural stability of these samples, as discussed previously. The wet samples show greater instability (larger dependence of the moduli on frequency), possibly due to the presence of water that acts as a plasticizer in the systems, making them more deformable.

Comparing the different samples (Table S4), incorporating PP in the formulations decreases *E*′_1_, although it has a more solid structure (less porous) with a greater cross-linking degree (Table S2). The result suggests that the cell walls of those extruded samples having lower PP generate a more consolidated structure that is more difficult to deform plastically. The addition of PP also generates a decrease in critical strain (the last strain in the viscoelastic range), further demonstrating the lower elastic stability with PP. GEN and GO generally promote the increase in *E*′_1_ and critical strain values. These results are consistent with those obtained from the static tensile test (Figure 8). The reference PUR pad has a considerably lower elastic modulus than the herein-produced materials, probably due to its more flexible structure. The wet samples showed a decrease in their moduli by 2 orders of magnitude, from 10^4^–10^5^ to 10^2^–10^3^ kPa (see Figure 9d–f), compared to the dry extrudates. Here, the critical strain of the wet systems was also higher than that of dry ones (Table S4), and the values approach those of the PUR pad, which did not alter its rheological parameters despite being soaked in water. The results corroborate the role of the water acting as a plasticizer in biobased materials, increasing the mobility of the chains and, therefore, reducing the rigidity of the systems.^43^

### 3.4 Bioactivity

#### 3.4.1 Hemocompatibility

**Figure 10:**
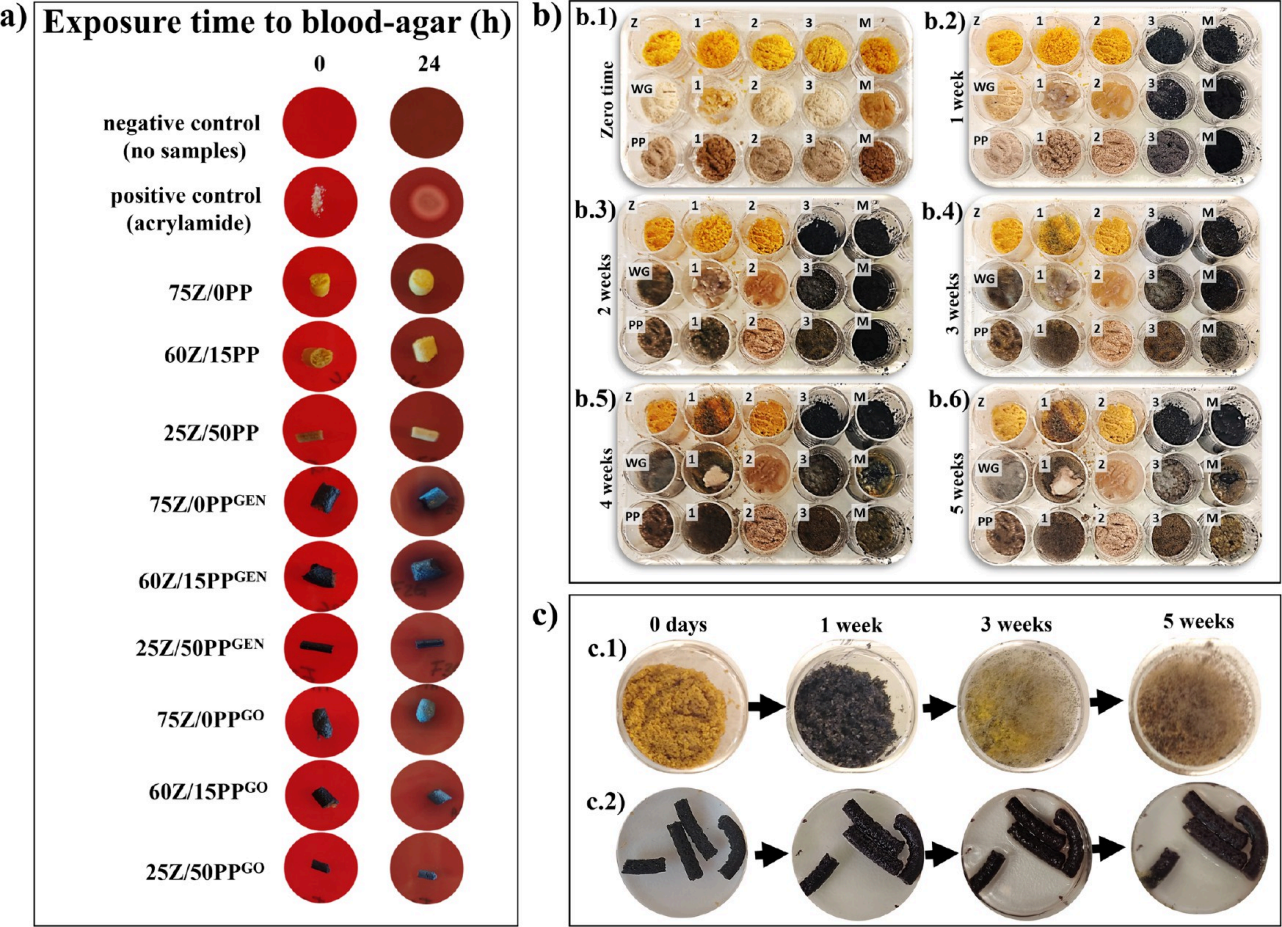
Noncytotoxic evidence of the samples obtained by reactive extrusion in direct contact with the agar/blood system, after incubation at 37 °C for 24 h and in the presence of air flow and 5% CO_2_ (a). Behavior of each of the proteins (Z, WG, PP) alone, with glycerol as plasticizer (1), with SBC (2), with GEN (3) and the mixture of all additives (M) exposed to 100% relative humidity as a function of time: *t* = 0 (b.1), 1 week (b.2), 2 weeks (b.3), 3 weeks (b.4), (e) 4 weeks (b.5), and 5 weeks (b.6). Nonextruded 40Z/35PP^GO^ exposed to 100% RH showing the presence of mold (c.1) compared to the extruded 40Z/35PP^GO^ sample (c.2).

The formulations with the highest porosity and swelling capacity (i.e., 75Z/0PP and 60Z/15PP with/without cross-linkers) and the formulation with the lowest porosity and highest limonene uptake (i.e., 25Z/50PP with/without cross-linkers) were selected for this test. Figure 10 shows that after 24 h, the whitish halo characteristic of cytotoxicity was not observed in any of the selected samples. However, the blood agar system in contact with the 75Z/0PP^GEN^ and 60Z/15PP^GEN^ samples showed darkening of the surface. In contrast, the blood agar system in contact with similar formulations cross-linked with GO (75Z/0PP^GO^ and 60Z/15PP^GO^) did not show any darkening of the blood. The darkening of the blood may be associated with an oxidative process caused by the presence of noncross-linked genipin molecules at the surface of the extruded samples leaching into the blood agar. On the contrary, the low content of genipin in the GO (ca. 14%) could have led to more reacted genipin, resulting in the absence of darkening of the blood.

#### 3.4.2 Antimicrobial Activity

The extruded samples did not exhibit any antimicrobial effect in the agar diffusion method. However, the antimicrobial activity against *S. aureus*, *P. aeruginosa*, and *G. candidum* was evident when determined by the microdilution method (Table 2). Overall, the samples showed antimicrobial activity to different extents and concentrations against *S. aureus*, *P. aeruginosa*, and *G. candidum* compared to the reference sample, showing no activity. For example, 75Z/0PP showed substantial activity against *S. aureus* and *P. aeruginosa* at 750 and 1000 μg/mL, respectively. On the other hand, its efficacy against *G. candidum* was lower at the same concentrations. The absence of inhibition zones in the agar, alongside the results in the microdilution method, is likely related to the different diffusion mechanisms of the antimicrobial components from the extruded samples in the herein-used methods. The disc diffusion assay requires the antimicrobial components to migrate to the surface of the sample, thereby creating an inhibition ring. The result indicates likely a stronger aggregation of the components in the extruded samples (e.g., genipin), concentration effects, interaction with the medium, and differences in microbial growth patterns in both tests.^44^

**Table 2:** Minimum Inhibitory Concentration (MIC) values of Films against *S. aureus, P. aeruginosa,* and *G. candidum*

samples	*S. aureus* (μg/mL)	*P. aeruginosa* (μg/mL)	*G. candidum* (μg/mL)
negative control	–ve	–ve	–ve
antibiotics	0.3 (Oxacillin)	0.3 (Ceftazidime)	
pad	1000	750	1250
75Z/0PP	750	1000	1000
75Z/0PP^GEN^	500	1250	750
75Z/0PP^GO^	1000	500	500
25Z/50PP	1000	500	1000
25Z/50PP^GEN^	1250	500	1500
25Z/50PP^GO^	1000	750	1250

The outcome does not undermine the future use of extruded and cross-linked protein-based porous materials because, in applications such as sanitary pads, the exposure time of these materials is short, thus minimizing the risk of bacterial growth. Also, the respective rest performed in a pad showed similar activity to the protein-based materials (Table 2). When the material is disposed of and biodegradation begins, the bioactive molecules will be released, allowing for the control of pathogenic bacteria during this process, avoiding harmful conditions for related working environments.^45^

#### 3.4.3 Mold Resistance Test

Figure 10b shows the visual inspection of each protein powder used, the effect of the different components added, and the respective mixtures when exposed to 100% RH. The first evidence of mold growth was observed after the second week in WG and PP powder with and without glycerol. However, these systems did not show mold when containing GEN, GO, and/or SBC (Figure 10b.3). Figure 10b.4 shows that mold grew on Z powder after 3 weeks when containing glycerol, while no visual evidence of mold is observed in the pure Z even after 5 weeks (Figure 10b.6). The results indicate the role of zein as a component aiding to increase the storage properties of future green porous absorbents.

The rapid mold growth in the samples with PP is related to the greater presence of carbohydrates (between 2 and 5% higher than in the WG), which is the main source of carbon for microbial growth.^46^ Likewise, glycerol typically increases mold growth since it has a high capacity to reduce its carbon atoms and become food for microorganisms.^47,48^ Here, no signs of mold are apparent in any protein powder containing SBC, even after 5 weeks (see Figure 10b). SBC has been reported to widely inhibit mold growth,^49,50^ especially green mold that forms on citrus fruits.^50^

Figures S7 and 10c show the behavior of the protein mixtures used to prepare the porous materials before extrusion (Table 1) and after being extruded. The protein mixtures and the extruded product (with and without GEN/GO) showed no evidence of mold growth after 5 weeks of exposure to 100% RH, irrespective of the amount of PP used (see Figure S7a,c,e,g). Only 40Z/35PP^GO^ powder mixture showed mold growth after 3 weeks of exposure (Figure 10c.1). However, Figure 10c.2 shows that this formulation did not show extensive mold even after 5 weeks of exposure after being extruded. The results indicate that the microorganism interaction with the samples depends not only on the protein/formulation component but also on their thermal history.

While biologically based materials potentially offer a more environmentally friendly alternative, one of their limitations is storage stability. However, the mold resistance of these materials demonstrated excellent storage properties for the formulations as powder and in extruded form. This is relevant for ensuring the quality of the formulations before their thermal processing in a future production line and once the material is produced and stored. It is worth mentioning that all extruded samples had a wet appearance already after 1–2 days of exposure at 100% RH. Such results show the potential of the materials in unexplored areas, such as biobased moisture absorbents in food packaging.

#### 3.4.4 Biodegradation and Bioassimilation

**Figure 11:**
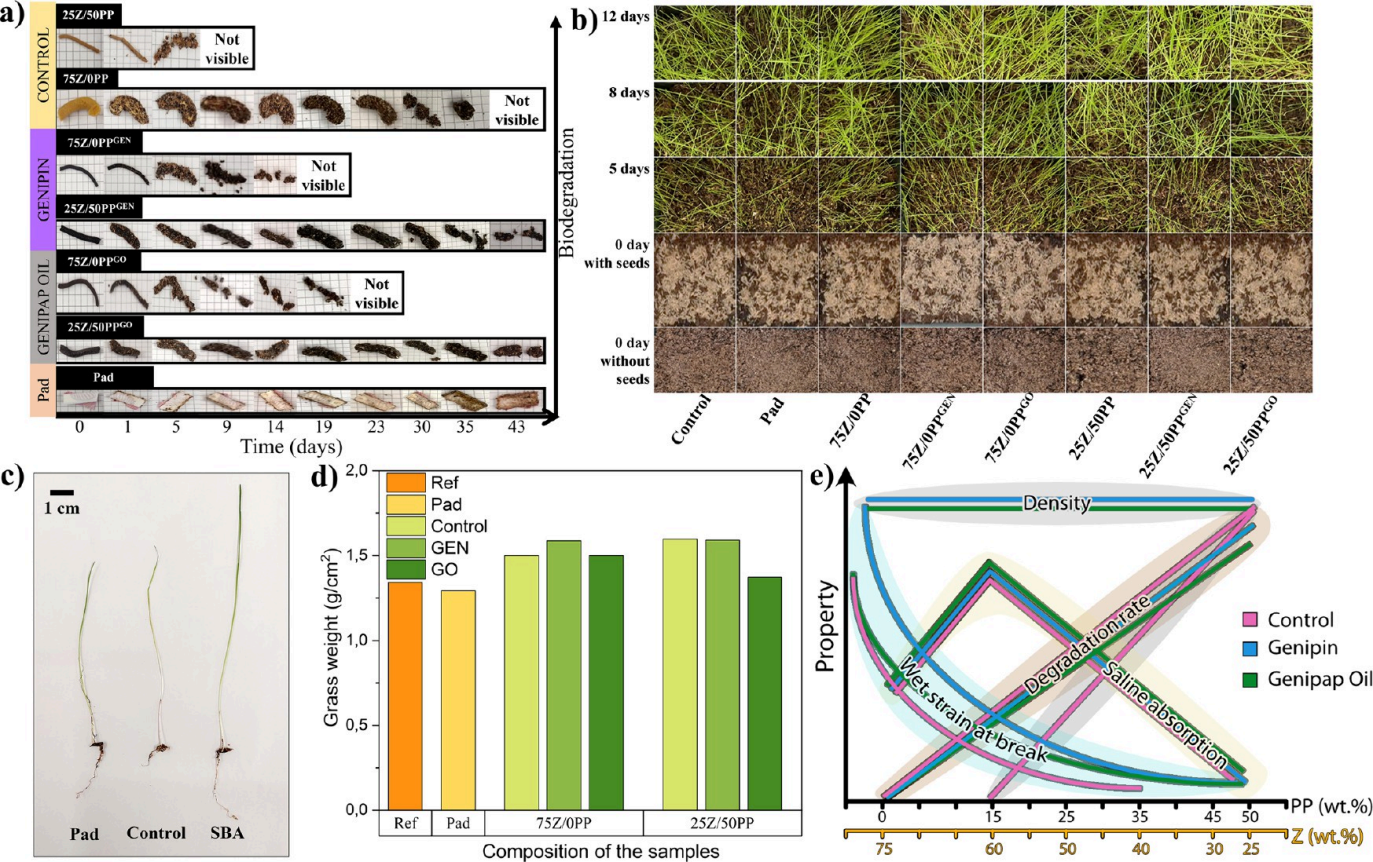
Soil biodegradability of 75Z/0PP and 25Z/50PP extruded samples (control, genipin, and genipap oil) for 43 days (a). Preliminary ecotoxicity study of the selected extrudates and the controls buried in soil with grass seeds after different time periods: top view of grass growth (b), comparison of representative grass leaves and roots of the commercial Pad, Control (no soil) and extruded samples SBA (c), and total grass weight (yield) for the different systems (d). Relationship between the PP:Z protein ration, the systems produced, and their structure-properties correlations e).

The visual appearance of the samples during soil biodegradation is shown in Figure 11a. The results show that noncross-linked samples biodegrade faster than cross-linked samples. The addition of PP influenced the biodegradation and resulted in all systems degrading in less than 20 days. Furthermore, the extruded samples with smaller diameters were found to degrade faster, with size being an important factor in degradation time.^51^ On the other hand, cross-linked samples form a three-dimensional network that is more resistant to biodegradation.^52^ It is important to highlight that all protein-based samples showed rapid and large biodegradation compared to that of the reference commercial pad, which did not present any evidence of degradation after completing the study.

To ensure that the degradation of the herein materials and their leachates does not impact ecosystems, we performed a preliminary soil toxicity study to assess if any potential contaminants inhibit regular plant growth (using grass seeds as in previous studies).^53^Figures 11b and S8 show that all systems had similar grass growth with respect to the control (only soil) and the Pad (Figure 11b). Additionally, the grass yield weight (leaf + root) is comparable for all of the studied samples (Figure 11c−d). This proof-of-concept reveals no immediate toxic effect when the tested SABs are discarded in the natural environment, and utilization pathways should be explored further, including relevant statistical analysis and different plant growth monitoring strategies. Here, the overall aspect of the grass after being removed from the soil suggests that grown on soil, including the biobased SABs, possess longer leaves and roots (see representative specimens in Figure 11c). The results indicate a possibility to use the leftovers from the material production as, for example, biostimulants in horticultural crops and should be explored in future studies, which is of interest for zero-waste production in the future. Figure 11e illustrates the relationship between the composition and properties of the prepared porous materials depending on the use of GEN and GO as cross-linking agent**s** and the amount of PP wt.%. The chart serves as a guide to select the desired composition and system depending on the property of interest, with each group in a different cluster.

## 4 Conclusions

Protein-based porous absorbent structures with high absorption capacity, improved mechanical properties, rapid biodegradation, and no ecotoxicity were prepared by reactive extrusion using genipin-rich oil as a cross-linking agent. The mechanical properties of the samples cross-linked with genipin oil were higher than reference materials and comparable to those where expensive purified genipin is used, despite the GO having low genipin concentration. In addition, incorporating the genipap oil resulted in the plasticization of the protein matrix due to the lipids contained in genipap oil. The results revealed the potential of the genipap oil to act not only as a cross-linking reagent but also as a processing aid. The use of the oil allows for better dispersion in the protein mixtures than commercial genipin powder (melting at temperatures above 110 °C) while allowing efficient in situ cross-linking of the material during extrusion. Despite the higher degree of cross-linking of the samples, extensive degradation activity in a soil environment was observed after 43 days, with commercial samples not showing any signs of soil degradation. The extrudates also have no impact on the germination and growth of grass seeds (used as a reference plant), indicating that the samples have low toxicity during biodegradation and could cope with scenarios in which the materials are disposed of in the natural environment. Finally, this work demonstrates the possibility of creating renewable-based materials that are competitive with commercial superabsorbent foams, reducing the pollution generated by their use in hygiene products, and opening new fields of study, such as their use as biostimulants in plant growth or other applications where a high surface area that is not available is convenient (i.e., insulating porous materials and controlled release systems).

## Supporting Information Available

Cross-linking degree data (Tables S1 and S2); 3D tomography data (Table S3); mold growth test setup (Figure S1); DSC results of pre-extruded samples (Figure S2); FTIR spectra in the region between 1760 and 1720 cm^–1^ of all the samples (Figure S3); relationship between the ratio of Z to PP and saline solution swelling at 5 min (Figure S4); representation of the relationship between genipin concentration and Young’s moduli (Figure S5); dynamic tensile test data (Table S4); cyclic compression curves at the same compression strain interval (0–30%) (Figure S6); mold resistance of the formulations (Figure S7); and side view of the germination box for the samples’ bioassimilation tests (Figure S8) (PDF)
