# Supplementary material for: Genipap Oil as a Natural Cross-Linker for Biodegradable and Low-Ecotoxicity Porous Absorbents via Reactive Extrusion
Source: Biomacromolecules. 2024 Oct 25;25(12):7642–59. doi: 10.1021/acs.biomac.4c00883 (PMC11632661; doi:10.1021/acs.biomac.4c00883)
Supplement: Supplementary file 1 — bm4c00883_si_001.pdf [file bm4c00883_si_001.pdf]

Supplementary Information

Genipap oil as a natural cross-linker for biodegradable and low-ecotoxicity porous absorbents via reactive extrusion.

Liliana B. Hurtado[†,‡,*] Mercedes Jiménez-Rosado', Maryam Nejati[§], Faiza Rasheed[&], Thomas Prade'', Amparo Jiménez-Quero[§,#], Marcos A. Sabino[†], Antonio J. Capezza[‡,*]

[†]*Dept. of Chemistry, B[5]IDA research group, Simon Bolivar University, Caracas 89000, Venezuela*

[‡]*Fibre and Polymer Technology Department, KTH Royal Institute of Technology, Teknikringen 56, Stockholm, SE-10044, Sweden.*

'Dept. de Química y Física Aplicadas, Universidad de León, Campus de Vegazana, 24007 León, Spain

[§]*Department of Chemistry, KTH Royal Institute of Technology, AlbaNova University Centre, SE-106 91, Stockholm, Sweden.*

[&]*Department of Biotechnology, Faculty of Biological Sciences, Quaid-i-Azam University, Islamabad 45320, Pakistan.*

''Dept. of Biosystems and Technology, Swedish University of Agricultural Sciences, Box 190, 243 22 Lomma, Sweden.

[#] *Division of Industrial Biotechnology, Department of Life Sciences, Chalmers University of Technology, 412 96 Gothenburg, Sweden.*

- E-mail address: lbhc@kth.se, ajcv@kth.se

Table S1. Crosslinking degree of the samples due to the addition of PP, using 75Z/0PP (no crosslinker sample) as a 0% crosslinking reference.

Sample	Crosslinking degree (%)
75Z/0PP (REF)	*0
60Z/15PP	-36,9 ± 0,3
50Z/25PP	-34,32 ± 0,07
40Z/35PP	2,4 ± 0,1
30Z/45PP	56,83 ± 0,02
25Z/50PP	54,8 ± 0,1

The sample 75Z/0PP is the reference (0% crosslinking).

Table S2. Crosslinking degree of the samples with GEN and GO taking as reference the samples of the same composition but without crosslinker.

Sample	Crosslinking degree (%)
75Z/0PP	*0
75Z/0PP^{GEN}	-10,1 ± 0,5
75Z/0PP^{GO}	-1,5 ± 0,3
60Z/15PP	*0
60Z/15PP^{GEN}	25,3 ± 0,2
60Z/15PP^{GO}	41,1 ± 0,1
50Z/25PP	*0
50Z/25PP^{GEN}	37,9 ± 0,2
50Z/25PP^{GO}	46,70 ± 0,01
40Z/35PP	*0
40Z/35PP^{GEN}	3,7 ± 0,1
40Z/35PP^{GO}	29,3 ± 0,2
30Z/45PP	*0
30Z/45PP^{GEN}	-3,03 ±0,06
30Z/45PP^{GO}	10,61 ± 0,03
25Z/50PP	*0
25Z/50PP^{GEN}	42,01 ±0,04
25Z/50PP^{GO}	7,91 ± 0,08

* The samples that have 0 % Crosslinking degree were taken as a reference from each system (i.e., the results for 50Z/0PP^{GEN} and 50Z/0PP^{GO} were normalized against the 50Z/0PP value).

Table S3. 3D tomography parameters of the selected samples with and without GEN and GO.

System	Porosity (%)	Sphericity (%)	Flatness (%)
**75Z/0PP**	15	63 ± 21	29 ± 13
**75Z/0PP^{GEN}**	36	51 ± 27	33 ± 17
**75Z/0PP^{GO}**	45	51 ± 25	65 ± 26
**25Z/50PP**	21	31 ± 23	74 ± 22

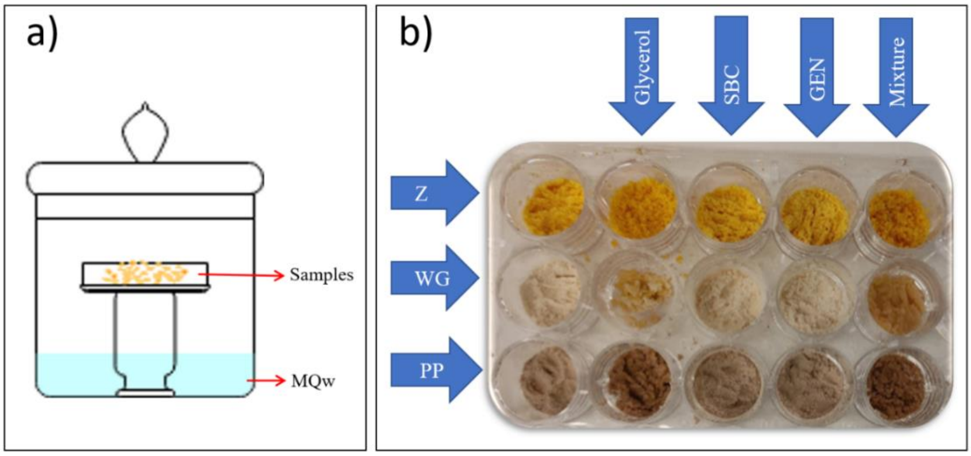

Figure S1. Mold growth test setup.

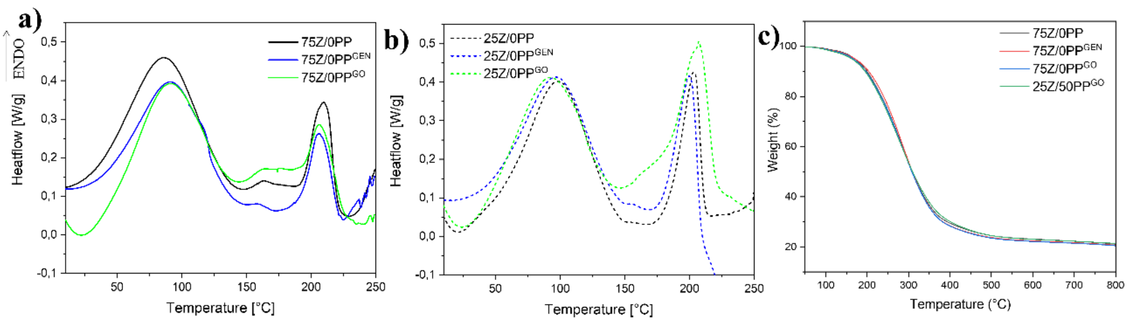

Figure S2. DSC results of pre-extruded samples: samples with ratio 75Z/0PP a) and samples with ratio 25Z/50PP b). TGA profiles of the porous materials after extrusion process c).

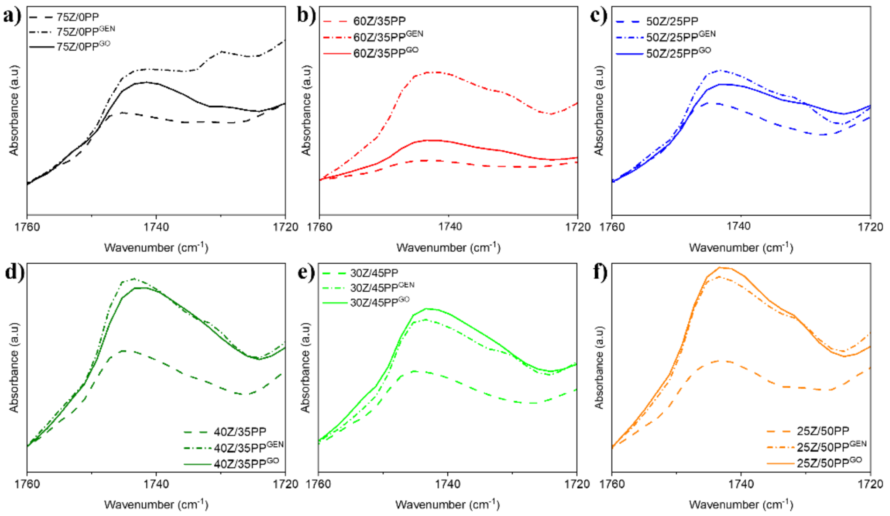

Figure S3. FTIR spectra in the region between 1760 and 1720 cm^{-1} of the formulations with the same protein composition and the presence of the crosslinking agent a) 75Z/0PP, b) 60Z/15PP, c) 50Z/25PP, d) 40Z/35PP, e) 30Z/45PP and f) 25Z/50PP.

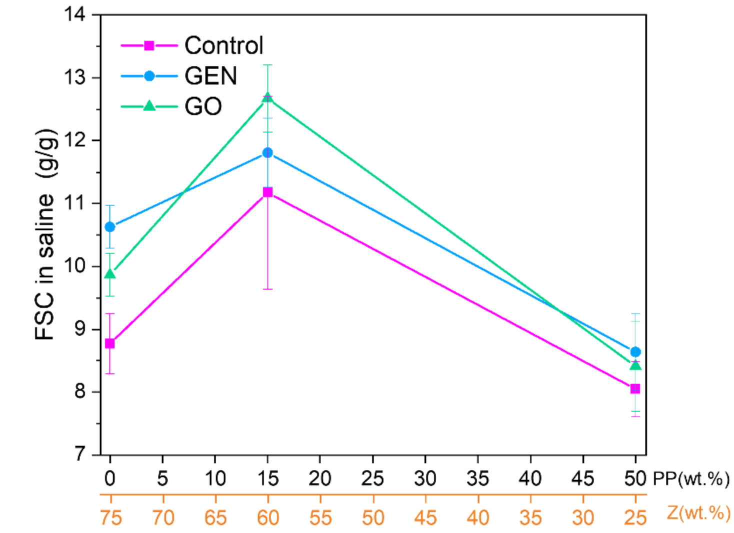

Figure S4. Relationship between the ratio of Z to PP and saline solution swelling at 5 min.

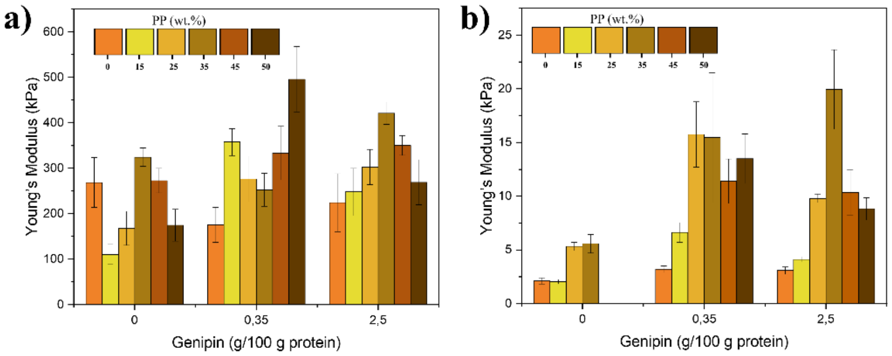

Figure S5. Representation of the relationship between genipin concentration and Young's moduli: dry samples a) and wet samples after being soaked in MQw for 24 h at 25 ºC b).

Table S4. Parameters obtained from the dynamic tensile test (Elastic Modulus at 1.0 Hz, E'; Loss Tangent at 1.0 Hz, tan δ; and Critical Strain) of dry and wet extruded samples.

Sample	DRY			WET		
	**E' (kPa)·10^{-3}**	**tan δ (-)**	**Critical Strain (%)**	**E' (kPa)·10^{-3}**	**tan δ (-)**	**Critical Strain (%)**
75Z/0PP	87.92 ± 2.48	0.166 ± 0.005	0.0087 ± 0.002	0.134 ± 0.033	1.207 ± 0.516	0.049 ± 0.006
60Z/15PP	37.95 ± 4.74	0.189 ± 0.012	0.0087 ± 0.002	0.172 ± 0.094	1.071 ± 0.345	0.169 ± 0.057
40Z/35PP	34.80 ± 7.15	0.227 ± 0.012	0.0103 ± 0.0065	0.215 ± 0.045	0.618 ± 0.514	0.116 ± 0.127
25Z/50PP	17.10 ± 0.92	0.298 ± 0.013	0.0022 ± 0.0008	0.567 ± 0.032	0.478 ± 0.106	0.115 ± 0.096
75Z/0PP^{GEN}	77.24 ± 13.9	0.162 ± 0.011	0.0142 ± 0.0119	0.192 ± 0.032	1.281 ± 0.266	0.068 ± 0.020
60Z/15PP^{GEN}	79.29 ± 2.39	0.171 ± 0.009	0.0139 ± 0.0002	0.260 ± 0.075	1.187 ± 0.265	0.084 ± 0.075
40Z/35PP^{GEN}	42.97 ± 7.11	0.215 ± 0.008	0.0110 ±0.0033	0.521 ± 0.080	0.657 ± 0.337	0.102 ± 0.027
25Z/50PP^{GEN}	54.57 ± 4.98	0.247 ± 0.008	0.0053 ± 0.0002	1.677 ± 0.701	0.404 ± 0.168	0.105 ± 0.034
75Z/0PP^{GO}	58.60 ± 7.60	0.158 ± 0.008	0.0153 ±0.0095	0.084 ± 0.025	1.17 ± 0.54	0.087 ± 0.058
60Z/15PP^{GO}	94.15 ± 3.75	0.174 ± 0.007	0.0154 ± 0.0092	0.304 ± 0.056	0.946 ± 0.314	0.131 ± 0.002
40Z/35PP^{GO}	51.49 ± 4.95	0.217 ± 0.009	0.0071 ± 0.0021	0.306 ± 0.087	0.522 ± 0.340	0.013 ± 0.004
25Z/50PP^{GO}	19.28 ± 2.96	0.268 ± 0.013	0.0084 ± 0.0004	0.765 ± 0.071	0.427 ± 0.210	0.004 ± 0.002
Pad	0.324 ±0.034	0.226 ± 0.071	0.00047 ± 0.0002	0.413 ± 0.099	0.291 ± 0.073	0.00049 ± 0.0002

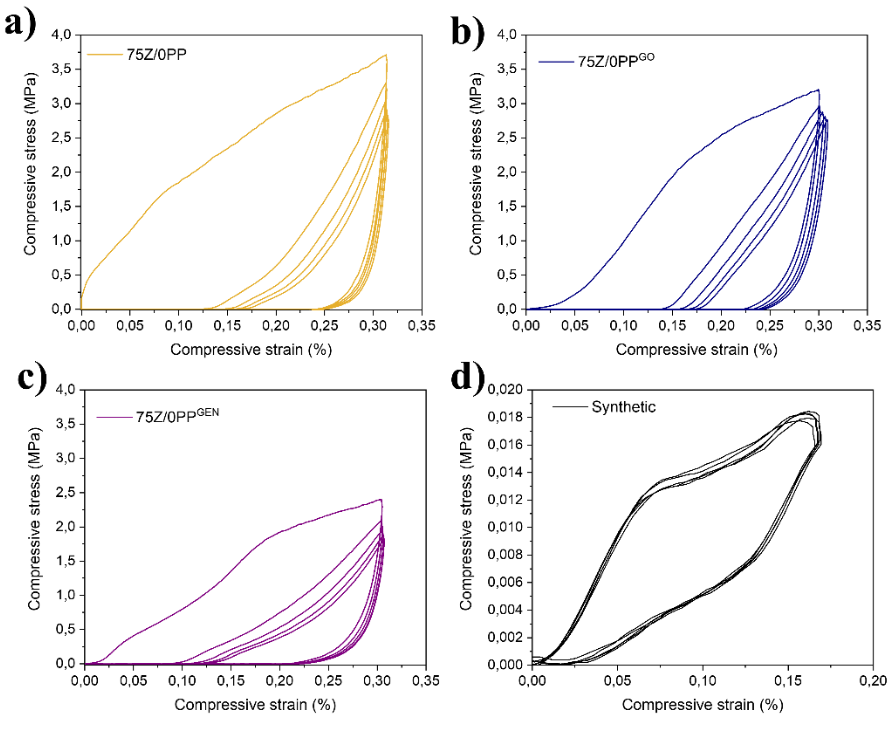

Figure S6. Representative cyclic compression curves at the same compression strain interval (0-30 %). Five compression cycles were performed.

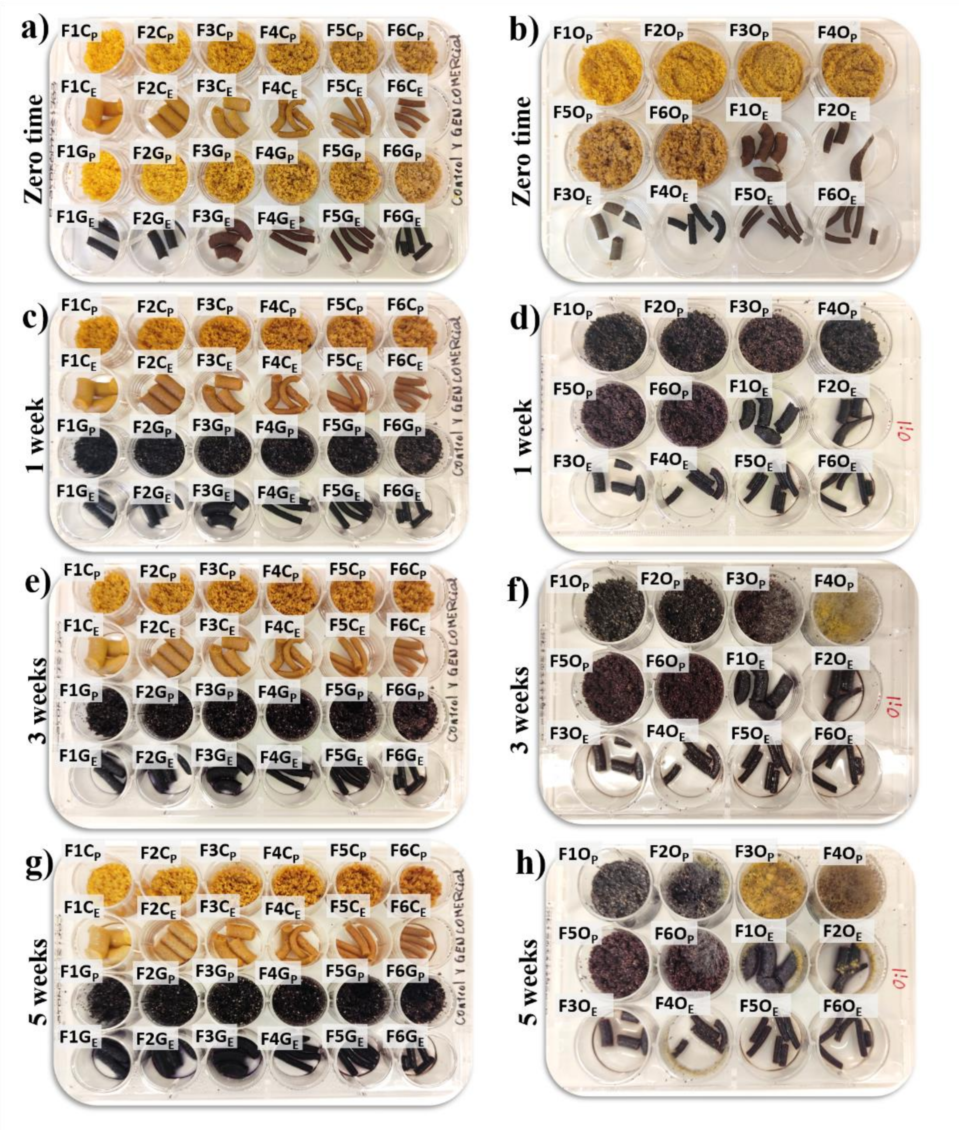

Figure S7. Mold resistance of the formulations developed in powder form (Pre-extruded, P) and the extruded formulations (E) for the control samples, with genipin (a, c, e and g) and genipap oil (b, d, f, and h) as a function of time. The mould resistance was explored by exposing the powders and their extrudates to 100% humidity at 25 ºC.

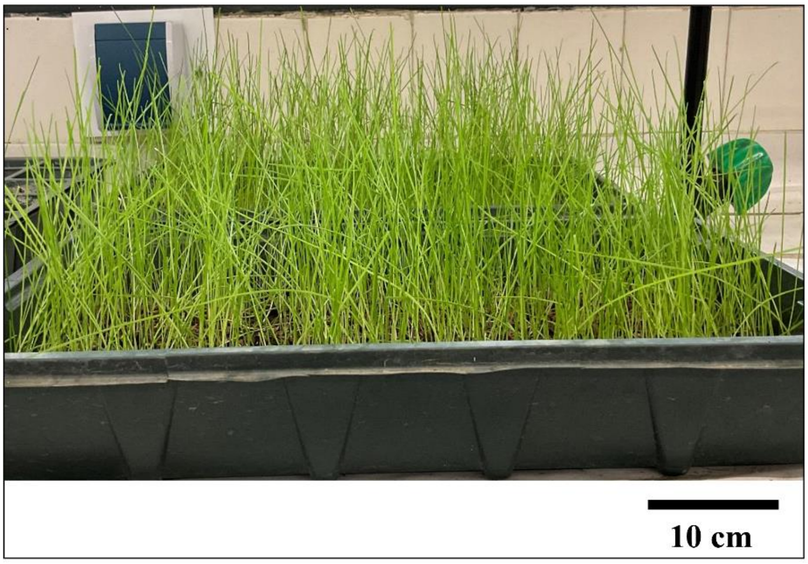

Figure S8. Side view of the germination box for the sample' bioassimilation tests using grass seeds for the control, reference synthetic pad, and porous protein-materials. The picture was taken after 12 days from planting the seeds.
